# Booster Vaccination Strategies for “Living With COVID-19”

**DOI:** 10.3389/fpubh.2022.896713

**Published:** 2022-06-03

**Authors:** Jung Eun Kim, Sunmi Lee, Hee-Sung Kim

**Affiliations:** ^1^Department of Mathematical Sciences, Ulsan National Institute of Science and Technology, Ulsan, South Korea; ^2^Department of Applied Mathematics, Kyung Hee University, Yongin, South Korea; ^3^Department of Internal Medicine, Chungbuk National University Hospital, Chungbuk National University College of Medicine, Cheongju, South Korea

**Keywords:** SARS-CoV-2, COVID-19, age-specific vaccination, booster shot strategies, comorbid-group priority vaccination, non-pharmaceutical intervention

## Abstract

Although the primary and secondary vaccination rates in Korea account for over 75% of the total population, confirmed cases of COVID-19 are dramatically increasing due to immune waning and the Omicron variant. Therefore, it is urgent to evaluate the effectiveness of booster vaccination strategies for living with COVID-19. In this work, we have developed an age-specific mathematical model with eight age groups and included age-specific comorbidities to evaluate the effectiveness of age-specific vaccination prioritization strategies to minimize morbidity and mortality. Furthermore, we have investigated the impacts of age-specific vaccination strategies for different vaccine supplies and non-pharmaceutical intervention levels during two periods: (1) when vaccine supply was insufficient and (2) after the emergence of the omicron variant. During the first period, the best option was to vaccinate the 30–49 year age group and the group with comorbidities to minimize morbidity and mortality, respectively. However, a booster vaccination should prioritize the 30–49 year age group to promote both minimal morbidity and mortality. Critical factors, such as vaccination speed, vaccine efficacy, and non-pharmaceutical interventions (NPIs), should be considered for effective vaccination prioritization as well. Primary, secondary vaccinations, and a booster shot vaccinations require different age prioritization strategies under different vaccination rates, vaccine efficacies, and NPI levels.

## 1. Introduction

SARS-CoV-2 infection has increased dramatically worldwide since the Omicron variant has become dominant. As of February 26, 2022, approximately 433 million cases and 6 million deaths had been reported worldwide (1). In Korea, the cumulative number of confirmed cases exceeded 2,800,000, and the death toll exceeded 7,000, causing a public health and economic crisis (2). Before the development of a coronavirus vaccine, most countries relied on non-pharmaceutical intervention strategies (NPIs), such as social distancing, isolation, contact tracing, and quarantine, as preventive measures against the spread of COVID-19.

Nonetheless, NPIs alone cannot end the epidemic, although they can slow the spread of the disease and prevent larger outbreaks to ensure that the rate of hospitalizations and deaths are manageable (3). However, lifting the NPIs could trigger a sharp rise in infection rate at any time without the majority of the population being immune to COVID-19, while the implementation of NPIs can cause economic damage and various adverse health effects (4, 5). Therefore, high vaccine coverage and NPI adherence are essential to control the COVID-19 pandemic. There is no clear evidence of which vaccination strategies are most effective in reducing the number of deaths and infections and enabling the safe lifting of NPIs without rebounding the infection.

There are two distinct situations in which South Korea must choose a vaccination strategy. The first is the period when the quantity of vaccine is insufficient at the initial stage of supply, and the second is the period when booster doses are recommended owing to waning of vaccine-induced immunity and the surge of the Omicron variant. In these two periods, when supply is insufficient to form herd immunity and the vaccination rate is low owing to vaccine hesitancy (6, 7), evidence is needed as to which population should be vaccinated first to effectively reduce both morbidity and mortality. Vaccination prioritization strategies for COVID-19 in Korea has involved vaccination of workers in high-risk medical institutions and epidemiological investigators, followed by vaccination of the high-risk group and the rest of the population, which was then expanded to target adolescents over 12 years of age with relatively low serious risks (8).

However, unlike the vaccination strategy in Korea, the World Health Organization's recommendations include immunocompromised persons, adults with comorbidities, and pregnant women in higher-priority use groups (9). Therefore, there is an urgent need to evaluate the effectiveness of prioritization vaccination for populations with comorbidities. In November 2021, when the domestic vaccination completion rate reached 75% and the primary vaccination rate reached 80%, the NPIs began to decrease. However, owing to the earlier-than-expected decline in the effectiveness of vaccines in the elderly (immunity waning), many patient deaths occurred that the medical system could not afford.

In addition, the Omicron variant that was announced on November 25, 2021, is expected to spread worldwide and become the dominant species in Korea during February 2022 (10). Caution is needed as the Omicron-variant virus may evade vaccine-induced or natural immunity to COVID-19 (11). A booster shot appears to counteract the waning protection of delta variants and can maintain the vaccine effect against Omicron variants (12). However, the effectiveness of booster vaccination may vary depending on the primary vaccination status of the entire population and current epidemic status. In addition, the effect of booster vaccination on the epidemic situation is determined by the proportion of Omicron in the total infection rate and the extent to which Omicron evades immunity (13).

In Korea, Omicron is expected to dominate in February 2022, but the booster shot rates remain relatively low at approximately 50% owing to safety concerns. Hence, we developed an age-specific mathematical model with eight age groups and included age-specific comorbidities to evaluate the effectiveness of age-specific vaccination prioritization strategies. In this study, we focused on the population age structure and underlying diseases of Chungcheongbuk-do (CB) province. We estimated age-specific transmission rates using age-specific demographics and confirmed case data of COVID-19 in CB. Furthermore, we investigated the impacts of age-specific vaccination strategies for different vaccine supplies and NPI levels during two periods: the first period was when vaccination began with insufficient supply, and the second period was after the emergence of the Omicron variant.

## 2. Methods

### 2.1. Epidemiological Data

As of February 26, 2022, South Korea had 2,831,283 confirmed COVID-19 cases and 7,895 deaths. Daily confirmed COVID-19 cases and deaths from April 1, 2020, to February 26, 2022, were obtained from the Korea Centers for Disease Control and Prevention (KCDC) and the CB provincial website (2, 14). The clinical and epidemiological characteristics of COVID-19 are heavily dependent on age; therefore, we incorporated age-specific features in our model, and age-specific cases were divided into eight groups, as shown in Table 1. The CB province comprises approximately 3% of the total Korean population, and COVID-19 cases in CB constituted approximately 2% of the total COVID-19 cases in South Korea. CB constitutes 7.4% of Korea; this implies that the population density per area is lower than the average in South Korea and, therefore, provides a rationale for the lower number of confirmed cases of COVID-19 in CB. However, the proportion of the elderly population (age > 50 years) in CB was higher than the overall proportion of the elderly population in South Korea (42 vs. 40%, respectively), whereas the proportion of younger age groups (age <30 years) was lower. Furthermore, the age-specific comorbidities in the CB province are presented in the last row of Table 1. Note that the population with comorbidities had at least one human immunodeficiency virus infection, tuberculosis, cancer, cardiovascular disease, chronic respiratory disease, chronic liver disease, diabetes, and chronic neurological disease (Table 1). The severity and case fatality rates are high in patients with comorbidities (15, 16).

**Table 1:** Age-specific population size and number of confirmed COVID-19 cases are compared for CB and South Korea.

		**0–9**	**10–19**	**20–29**	**30–39**	**40–49**	**50–59**	**60–69**	**70 -**
	Korea	3,874,174	4,746,103	6,754,283	6,788,072	8,220,344	8,606,589	6,957,802	5,735,658
Population	(51,683,025)	(7.50%)	(9.18%)	(13.07%)	(13.13%)	(15.91%)	(16.65%)	(13.46%)	(11.10%)
	(1,596,955)	(7.55%)	(9.24%)	(12.34%)	(12.06%)	(14.94%)	(16.92%)	(14.64%)	(12.33%)
	Korea	7,189	11,422	24,314	22,204	24,363	28,874	23,120	16,237
Confirmed	157,723	(4.56%)	(7.24%)	(15.42%)	(14.08%)	(15.45%)	(18.31%)	(14.66%)	(10.29%)
Cases	CB	114	199	464	482	498	650	475	341
	(3,222)	(3.54%)	(6.18%)	(14.4%)	(14.96%)	(15.46%)	(20.17%)	(14.74%)	(10.58%)
Population	CB	17,261	13,820	27,144	39,720	72,073	133,439	142,468	157,749
w/ comorbidities		(14.32%)	(9.37%)	(13.77%)	(20.63%)	(30.22%)	(40.39%)	(60.95%)	(80.13%)

*The percentage indicates the ratio of the population (confirmed cases) of each age group to the total population (confirmed cases). The age-specific population with comorbidities in the CB Province. The percentage indicates the ratio of population with comorbidities in each age group*.

**Figure 1:**
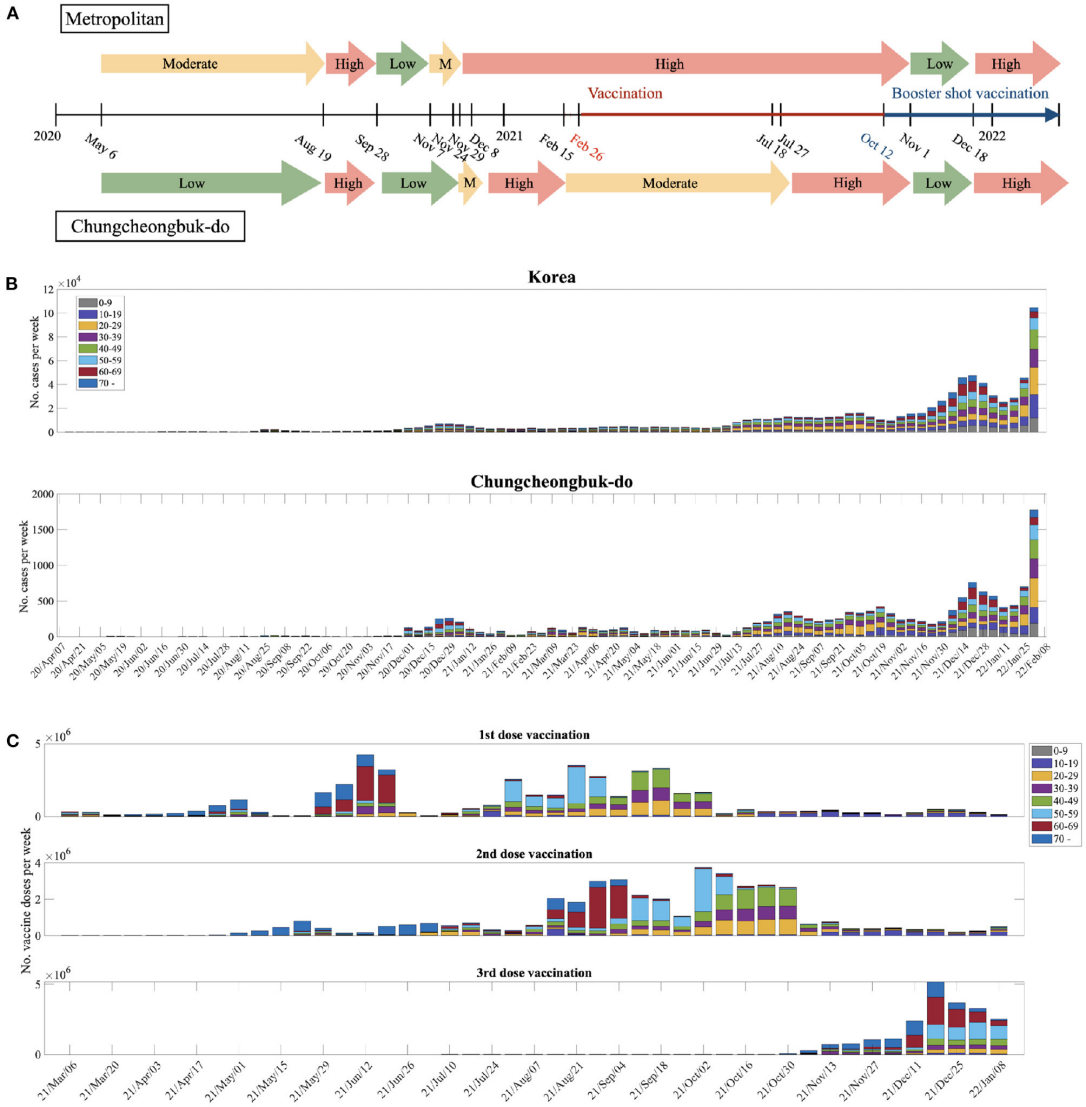
**(A)** Levels of NPI implemented by the Korean government. **(B)** Weekly age-specific data of confirmed COVID-19 cases across eight age groups are shown from April 1, 2020, to February 6, 2022. The top panel shows the weekly data of COVID-19 cases in South Korea, whereas the bottom panel shows the weekly data of COVID-19 cases in the CB province. **(C)** The 1st, 2nd, and 3rd vaccination doses per week for each age group in Korea.

Between January 2020 and January 2022, there were five large waves of COVID-19 in South Korea. Figure 1A compares the levels of NPI (social distancing) implemented by the Korean government in metropolitan and non-metropolitan areas. Note that a high level of social distancing was implemented at the end of November 2020 in metropolitan areas, and COVID-19 vaccination began on February 26, 2021. Figure 1B presents the weekly age-specific data of confirmed COVID-19 cases across eight age groups from April 1, 2020, to February 6, 2022. In Figure 1B, the top and bottom panels show the weekly number of COVID-19 cases in South Korea and the CB Province, respectively. In both panels, the weekly number of COVID-19 cases showed a similar age-specific temporal pattern. Lastly, Figure 1C shows the first, second, and third vaccination doses per week for each age group in Korea.

### 2.2. Mathematical Model

We developed an age-structured mathematical model to investigate the impact of age-specific vaccinations on COVID-19 transmission dynamics. The age-specific classes were composed of the following eight groups: 0−9, 10−19, 20−29, 30−39, 40−49, 50−59, 60−69, and 70−. The population was separated into eight compartments based on the epidemiological characteristics of each age group *i*. *S*_*i*_(*t*) is susceptible, *E*_*i*_(*t*) is exposed, *A*_*i*_(*t*) is unconfirmed infectious, *I*_*i*_(*t*) is confirmed infectious, $H_{i}^{m}\left(t\right)$ is quarantined or hospitalized with mild symptoms, $H_{i}^{s}\left(t\right)$ is hospitalized with severe symptoms, *R*_*i*_(*t*) is recovered, and *D*_*i*_(*t*) is dead. Moreover, $V_{i}^{F}\left(t\right)$ is the first dose vaccinated, $V_{i}^{S}\left(t\right)$ is the second dose vaccinated, $V_{i}^{B}\left(t\right)$ is the third dose (or booster) vaccinated, $R_{i}^{V^{F}}\left(t\right)$ is recovered and first-dose vaccinated, $R_{i}^{V^{S}}\left(t\right)$ is recovered and second-dose vaccinated, and we have the epidemiological status for vaccinated classes $X_{i}^{v}\left(t\right)$ at the same status as *X*_*i*_(*t*) for *X* = *E*, *A*, *I*, *H*^*m*^, *H*^*s*^, *R*.

Furthermore, we divided the total population into groups under normal conditions and groups with comorbidities. $X_{i}^{n}\left(t\right)$ and $X_{i}^{c}\left(t\right)$ are populations with normal conditions and comorbidities of the same status as *X*_*i*_(*t*) for *X* = *S*, *E*, *A*, *I*, *H*^*m*^, *H*^*s*^, *V*^*F*^, *V*^*S*^, *V*^*B*^, *E*^*v*^, *A*^*v*^, *I*^*v*^, *H*^*mv*^, *H*^*sv*^, respectively.

**Figure 2:**
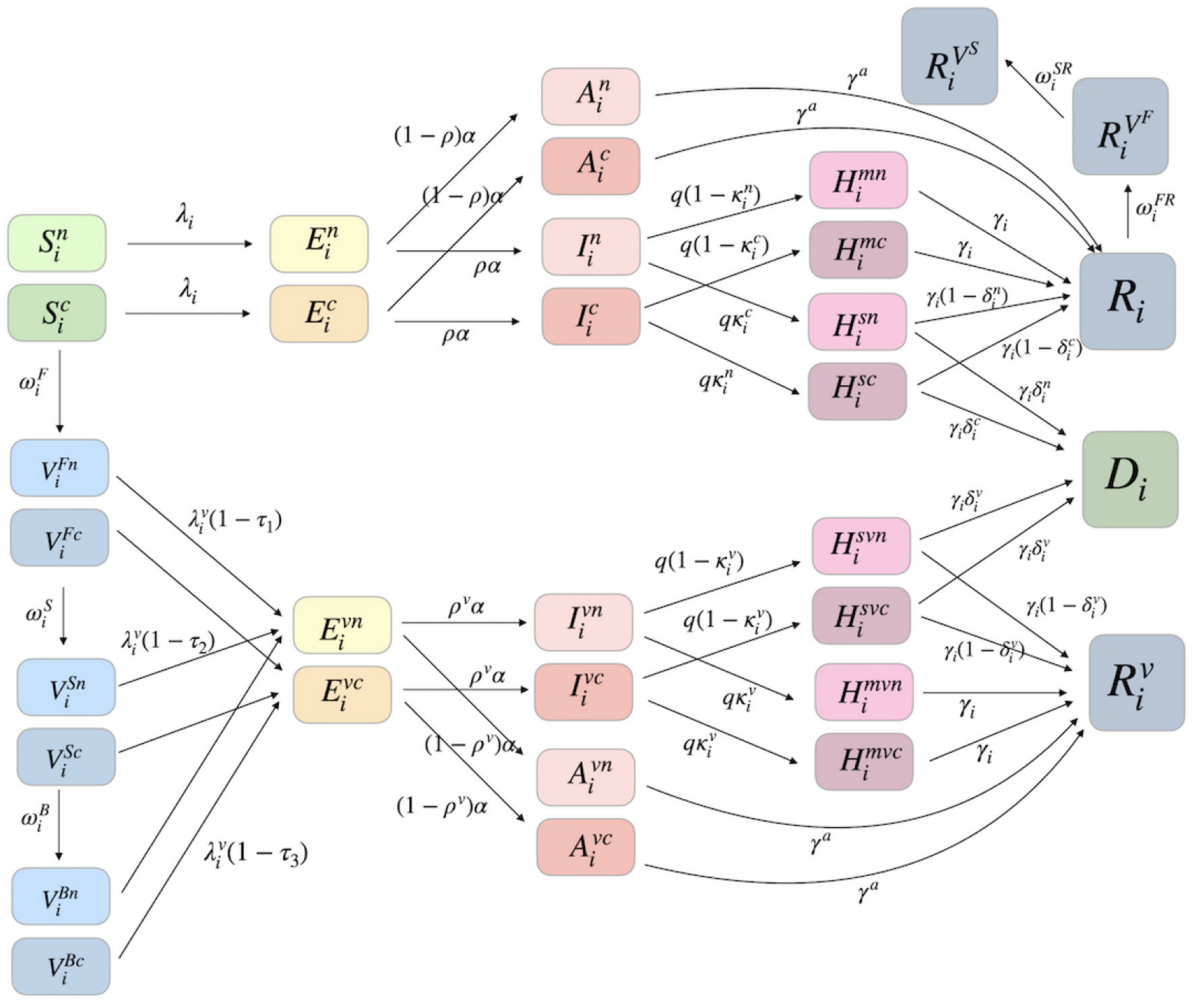
A schematic diagram of our age-specific mathematical model is shown in the presence of the first, second, and booster vaccinated classes. Furthermore, each age group is divided into two groups: the one under normal condition without comorbidities and the other one with comorbidities.

A schematic diagram of this model is shown in Figure 2. The model is presented as a system of ordinary differential equations, which are provided in Supplementary Section 1. The parameters used and the baseline values are shown in Supplementary Section 1. We computed the effective reproduction number, *R*_*t*_, which involves the fraction of susceptible population and potentially infectious vaccinated population. It measures the average number of secondary cases per infectious individual at time *t*, which is obtained by calculating the spectral radius of the next-generation matrix. The details of the derivation of *R*_*t*_ of the model are provided in Supplementary Section 2.

### 2.3. Age-Specific Vaccination Prioritization Strategies

In this subsection, we present four age-specific vaccination prioritization strategies. We have proposed four prioritization strategies, because there are critical age-specific characteristics, such as a higher activity level (aged 30–49 years) and a higher mortality rate (over 60 years and people with comorbidities). In addition, we considered a uniform vaccination strategy: all age groups to be vaccinated (over 20 years old) due to Korea's vaccination policy (only for people aged 19 years or older by October, 2021). These four strategies were applied to the primary dose and booster vaccination, except for the second dose vaccination. The second dose of vaccination was implemented after ψ days, the mean period between the first and the second dose of vaccination, as recommended by the Korean government.

- Strategy 1 (30−49): priority vaccination for those aged between 30 and 49 years.
- Strategy 2 (60+) : priority vaccination for those above 60 years old.
- Strategy 3 (*Comorb*.) : priority vaccination for those with comorbidities.
- Strategy 4 (20+) : uniform vaccination for those above 20 years old.

It was assumed that when the vaccination rate of the priority group reached 80%, vaccination was switched to the uniform vaccination strategy. Vaccination was stopped when the vaccinated population reached 80% of the total Korean population. At the beginning of the vaccination, Korea's policies were limited to 19 years or older; however, in October 2021, vaccination was extended to individuals older than 12 years. Therefore, for the first and second dose vaccination, people above 20 years of age were vaccinated before October 2021. The current vaccination data in Korea, according to which about 60% of those aged between 12 and 18 years were vaccinated, is reflected for the population $V_{i}^{Fn}$, $V_{i}^{Fc}$, $V_{i}^{Sn}$, and $V_{i}^{Sc}$. Currently, in Korea, the booster vaccination is implemented for aged 20s or older; hence, the booster shot is applied to people above 20 years of age. Then, the results are compared to the case when people above 10 years of age are vaccinated.

### 2.4. Estimation of Age-Specific Transmission Probability

In this subsection, we estimate the age-specific transmission probability β_*i*_ for each age group *i* per contact by fitting the age-specific confirmed case data in the CB. The contact matrix involving home, school, workplace, and other factors for each age group in Korea (17) was used and adjusted using the population in CB (18). The contact matrices *M*^*L*^, *M*^*M*^, and *M*^*H*^, for low, moderate, and high NPI levels, respectively, were constructed by the linear combination of location-specific matrices of home, school, workplace, and others and by multiplying the weights given in Table 2 based on the level of NPI implementation. The contact matrices are presented in Supplementary Section 3. The mobility of the vaccinated population was assumed to be higher than that of the unvaccinated population. Therefore, for the contact matrix of the vaccinated group, we used *M*^*L*^ for all NPI levels. The transmission probability $\beta_{i}^{v}$ of those vaccinated but without antibody in age group *i* per contact was assumed to be the same as that for β_*i*_.

**Table 2:** Age-specific transmission probability is estimated under different time periods for corresponding NPI levels.

**Vaccine**	**NPI**	**Time**	**Contact**	**Coefficient**	**{_β*i*_}_*i* = 1, ..., 8_**	***R*** **_*t*_**
**dose**	**level**	**interval**	**matrix**	**[home, school, work, others]**		
First	Low	10/31/2020– 11/19/2020	*M* ^*L*^	[1.1, 1, 1, 0.9]	0.1006, 0.0253, 0.0235, 0.0131, 0.0142, 0.0298, 0.1506, 0.0501	1.9220
Second	Moderate	2/25/2021–3/21/2021	*M* ^*M*^	[1.2, 0.5, 0.6, 0.8]	0.0242, 0.0159, 0.0380, 0.0228, 0.0118, 0.0159, 0.0747, 0.0859	1.2027
	High	1/5/2021–1/30/2021	*M* ^*H*^	[1.5, 0.2, 0.8, 0.6]	0.0338, 0.0135, 0.0183, 0.0202, 0.0147, 0.0218, 0.0470, 0.0375	1.0808
Third	Low	11/1/2021–12/1/2021	*M* ^*L*^	[1.1, 1, 1, 0.9]	0.0667, 0.0234, 0.1066, 0.0621, 0.0525, 0.0860, 0.6399, 0.5892,	1.2848

*The mean of R_t_ for each period is shown*.

Furthermore, we estimated the age-specific transmission probability under various NPI levels, as shown in Figure 1A. For the first and second dose vaccinations, {_β_*i*_}*i* = 1, ..., 8_ values were obtained under three different NPI levels: low, moderate, and high. For the booster shot, {_β_*i*_}*i*_ values were estimated based on the data from November 1 to December 5, 2021. This is due to the step-by-step recovery for the first major reorganization of the quarantine rules. The estimated {_β_*i*_}*i*_ values are presented in Table 2. Details of the age-specific estimation results are provided in Supplementary Section 4. We also carried out sensitivity analyses on parameters related to vaccination: β_*i*_, $\beta_{i}^{v}$, ρ, ρ^*v*^, τ_1_, τ_2_, ν_0_, and 1/ψ (see Supplementary Section 7).

## 3. Results

### 3.1. The Impacts of Age-Specific Vaccination Prioritization

**Figure 3:**
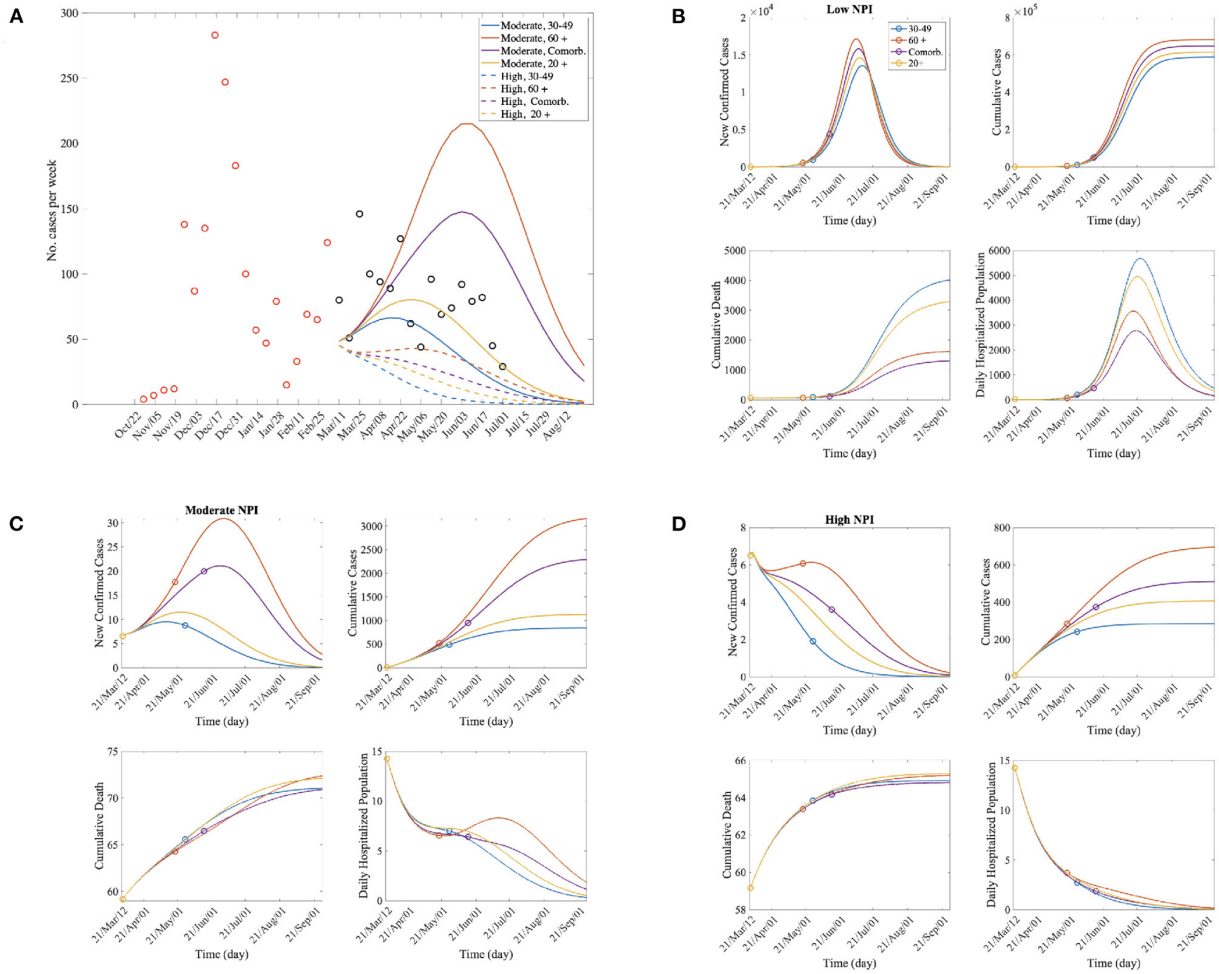
**(A)** The red and black circle represent the number of confirmed cases per week before and after vaccination, respectively. The number of confirmed cases is similar to the results obtained for the moderate NPI-level simulation. **(B–D)** Time series of new confirmed cases, cumulative confirmed cases, cumulative death, and daily hospitalized population for each vaccination priority scenarios; For each curve, the circle represents the end point of priority vaccination for a specific group according to the vaccination scenarios.

In this subsection, we investigate the impacts of age-specific vaccination prioritization for the primary and second doses from March 11, 2021 [14 days, duration for antibodies to be detectible (19), after the first vaccination began which is on February 26, 2021] to 6 months thereafter. The initial conditions were determined by reflecting the confirmed case data in CB, Korea at the start date of the simulation. Figure 3 shows the impact of the four age-specific vaccination prioritization strategies on daily confirmed cases and deaths. In Figure 3A, weekly age-specific confirmed COVID-19 cases of CB (circled) are compared with the model outputs under moderate and high NPI levels. The confirmed case data was most similar to the simulation results with a moderate NPI among the low, moderate, and high NPI scenarios (see the solid curves in Figure 3A). Indeed, according to Figure 1A, the level of NPI implemented in the CB region during that period was moderate.

The panels in Figures 3B–D show the time series of confirmed cases; cumulative confirmed cases; cumulative deaths; and the hospitalized population with severe symptoms at the low, moderate, and high NPI implementation levels, respectively. The number of confirmed cases was lowest when the 30−49 years age group was vaccinated first for all the NPI levels; however, the cumulative death was lowest when the comorbidity group was vaccinated first for all the NPI levels. When the comorbidity group was vaccinated first, both of the number of deaths and the number of confirmed cases were lower than when those over 60 were vaccinated first. Therefore, it can be said that it is an effective policy to inoculate the comorbidity group first in order to reduce the number of deaths and the patients with severe symptoms.

Epidemic outputs under the four age-specific vaccination prioritizations are summarized in Supplementary Tables 2–6. The number of confirmed cases decreased the most under Strategy 1 (30−49 years old) combined with a moderate NPI level. This implies that age-specific vaccination strategies with appropriate NPI policies are needed to maximize the benefits. In addition, we calculated the average *R*_*t*_ for 60 days under the vaccination priority scenarios in Supplementary Table 3. *R*_*t*_ decreased the most under Strategy 1 (30−49 years old). This is consistent with the results that the reduction in the number of confirmed cases.

Finally, we present the effects of the daily vaccination doses. Table 3 shows the number of cumulative confirmed cases and deaths under different NPI levels, daily dose of vaccination, and vaccination priority policies. Supplementary Table 2 shows percentage reduction of the estimated confirmed cases and deaths for each case. It can bee seen that at the Mod NPI level, the number of confirmed cases and the number of deaths due to vaccination were significantly reduced than at the low and high NPI levels. Therefore, if appropriate level of NPI policies such as social distancing are implemented, vaccine effectiveness can be increased. In all cases, the number of confirmed cases was the lowest when the 30−49 year olds were vaccinated first. When the daily dose is high (ν_0_ = 10, 000, 15, 000), that is, when the vaccine supply is sufficient, priority should be given to the comorbidity group to reduce the number of deaths. On the other hand, when a moderate or high NPI level with relatively small vaccination doses (ν_0_ = 5, 000, 7, 500), was implemented, the cumulative death was the lowest when the 30−49 year olds were first vaccinated. Moreover, when ν is small, the change in the number of cumulative confirmed cases and deaths according to the vaccination strategy is large. Therefore, it is important to apply an effective vaccination strategy when the vaccine supply is limited.

**Table 3:** The impacts of rollout speeds (daily doses of vaccine) on cumulative age-specific infected cases and deaths are shown under three different NPI levels.

	**NPI level**	**No vaccine**	**Daily doses**	**30−49**	**60+**	**Comorb.**	**20+**
		**(# of cases)**		**(# of cases)**			
Cumulative cases	Low	1.1290 ×10^6^	5,000	**834,970**	937,740	886,590	862,340
			7,500	**705,260**	812,590	768,470	733,410
			1,0000	**588,080**	681,520	648,010	613,990
			15,000	**399,660**	456,130	437,110	418,330
	Moderate	46,113	5,000	**2524.8**	17643	9337	3907.7
			7,500	**1287.9**	6953.1	4410.7	1847.2
			1,0000	**845.18**	3159.9	2289.9	1130.7
			15,000	**511.83**	1196.7	1012.4	631.51
	High	1876.7	5,000	**437.98**	1314.1	844.11	659.42
			7,500	**337.93**	934.99	639	496.66
			1,0000	**284.95**	694.3	510.53	406.17
			15,000	**228.32**	458.03	372.1	309.34
Death	Low	8655.6	5,000	6725.9	**3330.7**	3790.8	5709.8
			7,500	5432.7	2211.8	**2141.8**	4418.3
			1,0000	4019.6	1620.9	**1304.4**	3286.9
			15,000	1860.4	806.95	**596.75**	1556.4
	Moderate	320.28	5,000	**85.597**	106.88	96.668	92.241
			7,500	**75.142**	82.058	77.459	77.694
			1,0000	71.043	72.366	**70.9**	72.112
			15,000	67.811	66.863	**66.645**	67.996
	High	72.915	5,000	**65.93**	67.323	66.481	66.781
			7,500	**65.281**	65.985	65.385	65.835
			1,0000	64.922	65.195	**64.811**	65.288
			15,000	64.522	64.435	**64.236**	64.687

*Bold texts indicate the largest reduction*.

### 3.2. The Impacts of Age-Specific Booster Vaccination Prioritization

In this subsection, we investigate the impact of age-specific booster vaccination prioritization during the second period. As reported in recent studies, vaccination efficacy is decreasing due to the reduction in neutralizing antibodies (20) or the prevalence of the Omicron variant (21). Hence, we investigated the impact of the reduction in vaccine efficacy of the second dose (τ_2_), priority vaccination policy, and rollout speed for booster shots.

**Figure 4:**
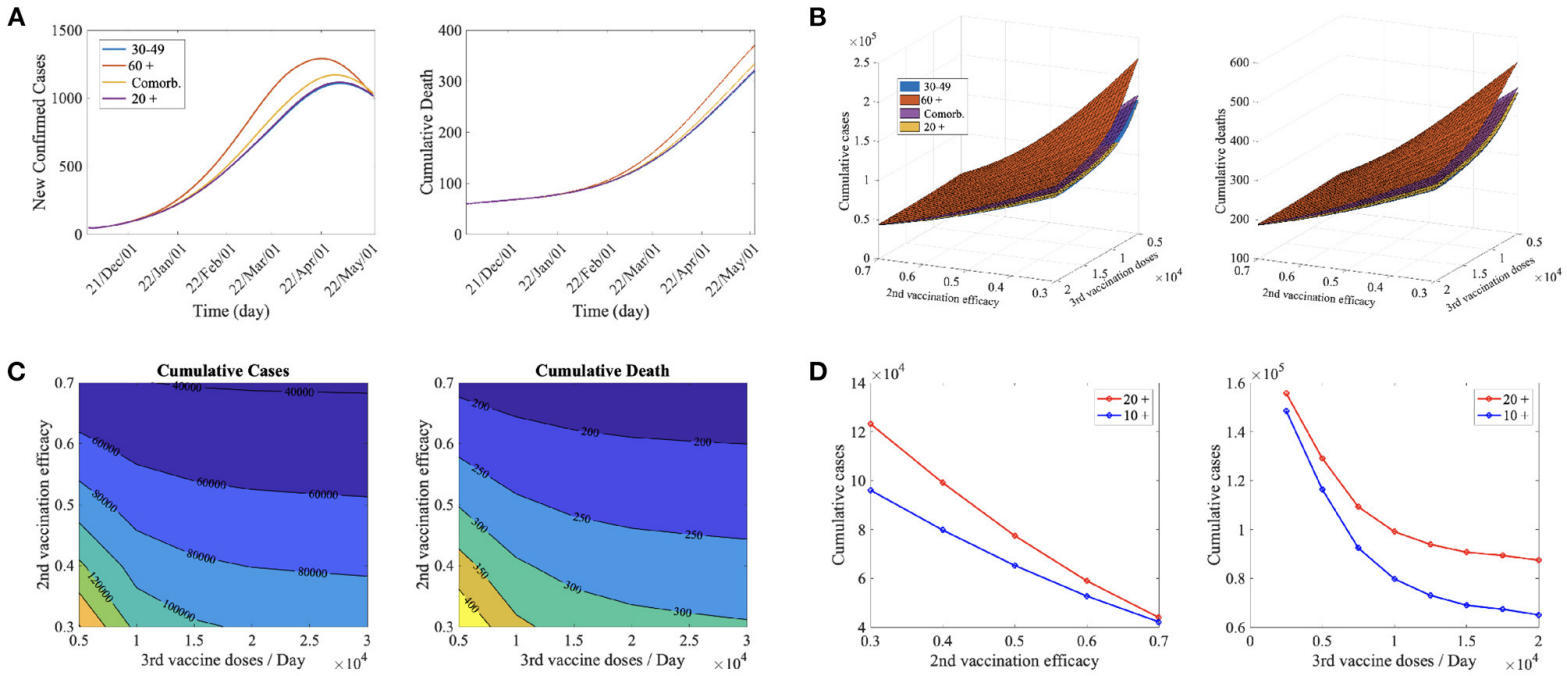
**(A)** Time series of confirmed cased and cumulative deaths for the priority vaccination policies (30–49, 60+, Comorb., 20 +) for the second vaccine efficacy, τ_2_ = 0.4, and daily the third vaccine dose, ν^*B*^ = 10, 000. **(B)** Cumulative confirmed cases and cumulative deaths for the priority vaccination policies (30–49, 60+, Comorb., 20 +) for ν^*B*^ = 5, 000, ..., 20, 000, and τ_2_ = 0.3, ..., 0.7. **(C)** Cumulative confirmed cases and cumulative deaths for the priority vaccination on those aged 30–49 years for ν^*B*^ = 5, 000, ..., 20, 000, τ_2_ = 0.3, ..., 0.7. **(D)** Comparison of cumulative confirmed cases for vaccination on 20 years and older (20 +) and 10 years and older (10 +) for (left) τ_2_ = 0.3, ..., 0.7, ν^*B*^ = 10, 000 and (right) τ_2_ = 0.4, ν^*B*^ = 2, 500, ..., 20, 000.

Figure 4A presents model outputs of daily confirmed cases (left) and cumulative death (right) according to the priority vaccination strategies with daily confirmed data where black and red circles represent the number of confirmed cases in CB before and after the Omicron is dominant in Korea (January 24th, 2021). The confirmed case increases rapidly after the Omicron variant becomes dominant. According to (20), the vaccination efficacy against infection is reduced to about 0.4 after 5 months of vaccination. Therefore, we assume that the vaccine efficiency is further reduced.

Figure 4A shows the effects of the priority vaccination policies (30−49, 60+, Comorb., 20+) under τ_2_ = 0.4, and daily third vaccine dose (ν^*B*^ = 10, 000). When those aged 30–49 years were vaccinated first, the number of confirmed cases and deaths decreased the most. The infection prevention rate decreases significantly while the death prevention rate does not decrease significantly (20), so prioritizing the high-activity group rather than the high-risk group seems to be effective in decreasing both confirmed cases and deaths.

Figures 4B,C shows the cumulative confirmed cases (left) and cumulative deaths (right) for 180 days in the variation of second vaccination efficacy (τ_2_) and daily third vaccine doses (ν^*B*^) for (Figure 4C) the priority vaccination strategies policies (30−49, 60+, Comorb., 20+) and (Figure 4D) for those aged 30−49 years. In Figure 4B, priority vaccination on 30−49 years groups and on 60+ years group are the most and the least effective strategies reducing cumulative cases and deaths in almost all cases. Figure 4C shows that as the second vaccination efficacy is smaller, the cumulative cases and deaths decrease more when the daily third vaccine doses increase. Therefore, it is necessary to accelerate the third vaccination as the efficacy of the second vaccination decreases.

In Korea, the first and second vaccinations are currently administered to teenagers, but booster shot vaccinations are not implemented in this subpopulation. We also studied the effects of booster shot inoculation on teenagers. Figures 4C,D show the difference in the number of confirmed cases when those aged 20 or older and those aged 10 or older were vaccinated according to the variation in second vaccination efficacy (Figure 4C) and the rollout speed of third vaccination (Figure 4D). For all cases, the cumulative confirmed cases were smaller when 10 years of age and over were vaccinated. It was shown that when the second vaccination efficacy was lower (Figure 4C) and the daily third vaccine dose was greater (Figure 4D), vaccination of teenagers reduced the number of confirmed cases. Currently, Korea's secondary vaccine efficiency is decreasing owing to the prevalence of Omicron, and the vaccine supply is sufficient. Therefore, it is recommended for teenagers to be vaccinated with a booster shot.

### 3.3. The Impacts of Different NPI Levels

In this subsection, we illustrate the impact of the mitigation of NPI levels combined with age-specific vaccination for the primary and second doses (before booster shots). As the vaccination rate increased rapidly, the government planned to relax the NPI policy, and recently, the number of confirmed COVID-19 cases has increased significantly. Hence, we performed simulations of NPI relaxation from moderate to low NPI levels. We compared the effectiveness of the NPI-level mitigation policy for a population of individuals older than 10 and 20 years.

**Figure 5:**
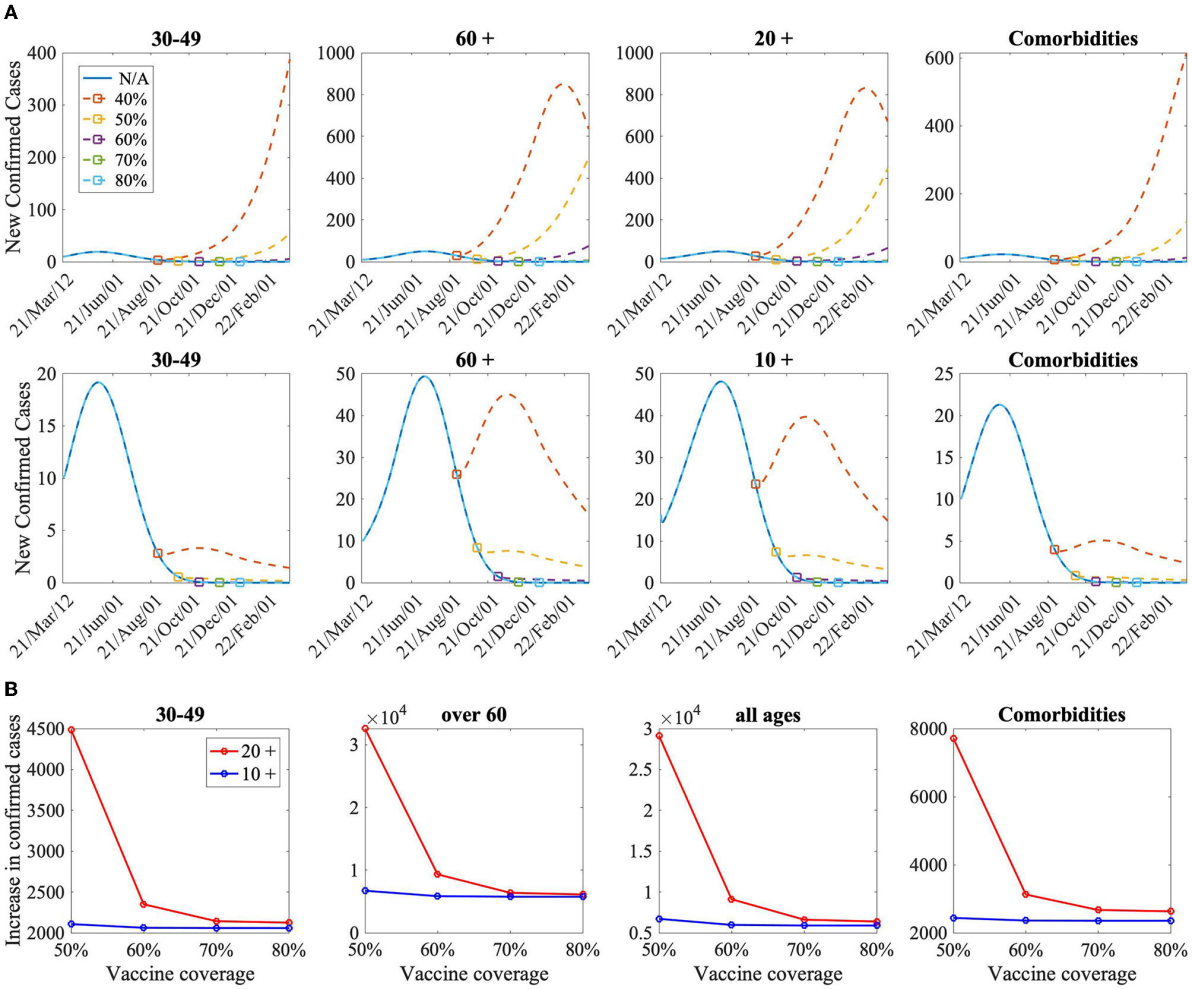
**(A)** The time series of the number of new confirmed cases according to the reduction of the NPI level for the secondary vaccination rates (50–80%). N/A indicates no mitigation of NPI level for the simulation duration. The top and bottom panels represent the case of vaccination-prioritization strategies for individuals aged 20 years and more and aged 10 years and more, respectively. **(B)** Comparison of increase of cumulative confirmed cases when vaccination is implemented for aged 20 and older (20 +), and for aged 10 and older (10+).

Figure 5A shows the time series of the number of new confirmed cases when the NPI level is mitigated based on the given secondary vaccination rates when the vaccination is implemented on those aged 20 years and older (top panels) and on those aged 10 years and older (bottom panels). The increase in the cumulative confirmed cases under the mitigation of NPI level when the vaccination is implemented on individuals aged 20 years and older (in red curves) and 10 years and older (in blue curves) are compared in Figure 5B.

It has been shown that too early NPI-level mitigation with low vaccination coverage could bring about another COVID-19 outbreak wave. Figure 5 shows that the increase in the confirmed cases was lower when those aged 10 years and older were vaccinated than when those aged 20 years and older were vaccinated, and the difference in the confirmed cases was greater when the vaccination coverage was low. Therefore, to mitigate NPIs effectively, it is necessary to vaccinate various groups of the population, not only to consider the vaccination rate.

## 4. Discussion

In this study, we developed an age-specific mathematical model to investigate the impact of vaccination allocation in combination with NPIs on COVID-19 transmission dynamics in two periods: when vaccination begins and when booster shots are needed owing to waning of vaccine-induced immunity. Our results indicate that at the initial stage of vaccination, a priority vaccination strategy for those with comorbidities was most effective for reducing mortality, regardless of the NPI level. However, a priority vaccination strategy for individuals aged 30−49 years was most effective in reducing morbidity.

Previous studies have revealed that COVID-19 patients with pre-existing comorbidities, such as hypertension, diabetes mellitus, chronic respiratory disease, malignancies, and HIV, could develop a life-threatening situation (22). Our data showed that a vaccine plan prioritizing a population with comorbidities is an effective alternative because, it can lower both mortality and morbidity compared with that for those aged 60 years and older. A modeling study evaluating the performance of the Centers for Disease Control in the United States showed that a higher prioritization of individuals with comorbidities led to better outcomes compared to the current vaccine allocation strategies (23). In Korea, to prevent the collapse of the medical system and minimize the number of deaths, the first vaccination was planned for medical personnel, workers, and the elderly in nursing homes (8). In our study, it was suggested that if the inoculation of the high-activity group is delayed, the increase in the number of infections may place a burden on the medical system. However, the vaccine's transmission block effect is not as effective as its mortality reduction effect (24). Therefore, it is necessary to maintain a policy to reduce mortality by prioritizing the vaccination of those with comorbidities and the elderly, rather than younger age groups.

In our study, when NPI was relieved early in all vaccination scenarios, the number of infections increased, resulting in a shortage of medical resources. These results are consistent with studies suggesting that maintenance of NPIs is necessary to increase the effectiveness of vaccines and reduce the number of infected people (25, 26). By November 1, 2021, 75% of the population in Korea had been vaccinated. Such high vaccine coverage was expected to reduce mortality and severity of infection, and the government decided to ease the NPI. However, this period coincided with the waning of vaccine-induced immunity period of the vaccine, and the infection spread rapidly among the elderly in nursing homes and communities. As a result, the number of critically ill patients rapidly increased, and NPI relief was canceled 46 days after starting owing to an insufficient medical system for critically ill patients. According to a study in the UK, the timing of NPI mitigation should be decided according to the vaccine coverage. In addition, when a mutant virus appears, NPI mitigation should be performed to evaluate the effectiveness of the vaccine and vaccination rate (27).

Comorbidities are known to be independent risk factors for severe progression of COVID-19 infection (28). Although comorbidities associated with severe progression are predominantly evident in the elderly, the presence of comorbidities might worsen the prognosis of young and middle-aged COVID 19 patients (29). In a study evaluating the vaccination strategy of the US CDC, higher prioritization of individuals with comorbidities in all age groups improved outcomes compared to the CDC allocation (23). It is consistent with the results of this study that vaccination of people with comorbidities at any age can be an effective alternative to prevent infection and severe progression.

The importance of booster shot vaccination is increasing, because vaccine efficiency is reduced due to the waning of secondary vaccines coupled with the emergence of variants of concern, such as Omicron. Our results show that booster shots are effective when applied preferentially to high-activity groups rather than to high-risk groups. This is supported by a retrospective cohort study showing consistently high effectiveness of BNT162b2 against COVID-19-related hospital admissions and severe death (20, 30, 31). However, a recent report suggested that booster shots are effective in reducing severe COVID-19 related outcomes (32). Thus, long-term vaccine effectiveness data against severe COVID-19-related outcomes must be continuously monitored in Korea. It can be seen that the rollout speed of the booster shots becomes more important as the secondary vaccine efficiency decreases. Previous studies on various vaccine rollout scenarios also emphasize the importance of rapid vaccine rollout to vulnerable populations and increasing coverage to avoid future surges (33, 34). In countries with limited infection-induced immunity, such as Korea, a rapid vaccine rollout strategy is needed to overcome the reduced vaccine efficacy against Omicron combined with its high transmissibility (13). It is also necessary to closely check whether the effectiveness of the vaccine is maintained in the population while mitigating NPIs.

In May 2021, the Pfizer-BioNTech COVID-19 vaccine was approved for expanded vaccination of individuals older than 12 years in the United States (35). On July 16, 2021, in Korea, the vaccine was approved for use in individuals older than 12 years of age as well. Based on the results of evidence-based research demonstrating the efficacy and safety of vaccination among adolescents, several countries are planning to vaccinate adolescents. However, as most adolescents are known to experience mild symptoms from COVID-19 infection and have few sequelae, questions remain as to whether the vaccine should be administered to children or should be provided to areas where the majority of at-risk groups have not been vaccinated. Nonetheless, as the rate of COVID-19 infection among adolescents is increasing owing to the Omicron variant, an increased vaccination rate among the elderly, in combination with the mitigation of the NPI level, is necessary to plan an effective vaccination strategy for adolescents (36). In our study, model-based analysis predicted that a vaccination strategy for teenagers would decrease the number of COVID-19 patients. Therefore, studies on the efficacy and safety of vaccines in domestic adolescents are needed.

This study has some limitations. First, for a decrease in vaccine efficacy due to waning of vaccine-induced immunity, sequential decrease in vaccine efficacy by age based on the priority vaccination strategy was not considered. In other words, the same vaccine efficacy was applied to all age groups. Second, the transmission rate parameter β was estimated using data from the period before the prevalence of Omicron variants, so there may be a difference from the current transmission rate. Also, different initial conditions and simulation duration might affect our results. Lastly, waning of vaccine-induced immunity is not explicitly modeled in study, therefore, this assumption may affect our results.

In conclusion, at the initial stage of vaccination, when the amount of vaccine is insufficient, the policy of vaccinating those with comorbidities or the elderly who are at a high risk of death will help reduce the number of deaths. Second, because sudden alleviation of NPI can cause a surge in infected patients, the level of NPI should be determined while closely evaluating whether vaccine coverage and effectiveness of the vaccine are maintained. Third, when vaccine immunity wanes, faster vaccination rollouts may reduce mortality rates. As Korea has limited infection-induced immunity across populations, it is necessary to closely monitor infection-induced and vaccine-induced SARS-CoV-2 immunity while deciding to open up and “live with COVID-19”.

## Data Availability Statement

The datasets presented in this study can be found in online repositories. The names of the repository/repositories and accession number(s) can be found below: the daily case data are publicly available from Ministry of Health and Welfare, South Korea (http://ncov.mohw.go.kr/).

## Author Contributions

Conceptualization and validation: H-SK and SL. Data curation: H-SK. Formal analysis, visualization, and Methodology: JK. Funding acquisition and writing—original draft: JK and H-SK. Writing—review and editing: JK, H-SK, and SL. All authors contributed to the article and approved the submitted version.

## Funding

This work was supported by a National Research Foundation of Korea (NRF) grant to JK and funded by the Korean government (MSIT) [grant number NRF-2021R1I1A1A01044426]. H-SK was supported by the research grant of the Chungbuk National University in 2021.

## Conflict of Interest

The authors declare that the research was conducted in the absence of any commercial or financial relationships that could be construed as a potential conflict of interest.

## Publisher's Note

All claims expressed in this article are solely those of the authors and do not necessarily represent those of their affiliated organizations, or those of the publisher, the editors and the reviewers. Any product that may be evaluated in this article, or claim that may be made by its manufacturer, is not guaranteed or endorsed by the publisher.
