# Supplementary material for: Booster Vaccination Strategies for “Living With COVID-19”
Source: Front Public Health. 2022 Jun 3;10:896713. doi: 10.3389/fpubh.2022.896713 (PMC9204168; doi:10.3389/fpubh.2022.896713)
Supplement: Supplementary file 1 [file Data_Sheet_1.PDF]

Supplementary Material

1 A MATHEMATICAL MODEL

The age-specific classes are composed of the following eight groups, $0-9$, $10-19$, $20-29$, $30-39$, $40-49$, $50-59$, $60-69$, and $70-$. The population is separated into eight compartments based on the epidemiological characteristics of each age group i. $S_i(t)$ is susceptible, $E_i(t)$ is exposed, $A_i(t)$ is unconfirmed infectious, $I_i(t)$ is confirmed infectious, $H_i^m(t)$ is quarantined or hospitalized with mild symptoms, $H_i^s(t)$ is hospitalized with severe symptoms, $R_i(t)$ is recovered, and $D_i(t)$ is dead. Moreover, $V_i^F(t)$ is first-dose vaccinated, $V_i^S(t)$ is second-dose vaccinated, $V_i^B(t)$ is third-dose (or booster) vaccinated, $R_i^{V^F}(t)$ is recovered and first-dose vaccinated, $R_i^{V^S}(t)$ is recovered and second-dose vaccinated, similarly, we have the epidemiological status for vaccinated classes $X_i^v(t)$ at the same status with $X_i(t)$ for $X = E$, A, I, H^m, H^s, R. Furthermore, we divide the total population into the groups in normal condition and the groups with comorbidities. $X_i^n(t)$ and $X_i^c(t)$ are populations with normal condition and comorbidities at the same status with $X_i(t)$ for $X = S$, E, A, I, H^m, H^s, V^F, V^S, V^B, E^v, A^v, I^v, H^{mv}, H^{sv}, respectively. The used parameters and baseline values are given in Table S1

$$
\begin{aligned}
\dot{S}_i^n &= -\lambda_i S_i^n - \omega_i^{Fn} \\
\dot{S}_i^c &= -\lambda_i S_i^c - \omega_i^{Fc} \\
\dot{E}_i^n &= \lambda_i S_i^n - \alpha E_i^n \\
\dot{E}_i^c &= \lambda_i S_i^c - \alpha E_i^c \\
\dot{A}_i^n &= (1-\rho)\alpha E_i^n - \gamma^a A_i^n \\
\dot{A}_i^c &= (1-\rho)\alpha E_i^c - \gamma^a A_i^c \\
\dot{I}_i^n &= \rho\alpha E_i^n - q I_i^n \\
\dot{I}_i^c &= \rho\alpha E_i^c - q I_i^c \\
\dot{H}_i^{mn} &= q(1-\kappa_i^n) I_i^n - \gamma_i H_i^{mn} \\
\dot{H}_i^{mc} &= q(1-\kappa_i^c) I_i^c - \gamma_i H_i^{mc} \\
\dot{H}_i^{sn} &= q\kappa_i^n I_i^n - \gamma_i(1-\delta_i^n) H_i^{sn} \\
\dot{H}_i^{sc} &= q\kappa_i^c I_i^c - \gamma_i(1-\delta_i^c) H_i^{sc} \\
\dot{R}_i &= \gamma^a (A_i^n + A_i^c) + \gamma_i (H_i^{mn} + H_i^{mc}) + \gamma_i((1-\delta_i^n) H_i^{sn} + (1-\delta_i^c) H_i^{sc}) - \omega_i^{FR} \\
\dot{D}_i &= \gamma_i(\delta_i^n H_i^{sn} + \delta_i^c H_i^{sc}) + \gamma_i \delta_i^v (H_i^{sv} + H_i^{sv}) \\
\dot{V}_i^{Fn} &= \omega_i^{Fn} - \lambda_i^v (1-\tau_1) V_i^{Fn} - \omega_i^{Sn} \\
\dot{V}_i^{Fc} &= \omega_i^{Fc} - \lambda_i^v (1-\tau_1) V_i^{Fc} - \omega_i^{Sc} \\
\dot{V}_i^{Sn} &= \omega_i^{Sn} - \lambda_i^v (1-\tau_2) V_i^{Sn} - \omega_i^{Bn} \\
\dot{V}_i^{Sc} &= \omega_i^{Sc} - \lambda_i^v (1-\tau_2) V_i^{Sc} - \omega_i^{Bc} \\
\dot{V}_i^{Bn} &= \omega_i^{Bn} - \lambda_i^v (1-\tau_3) V_i^{Bn} \\
\dot{V}_i^{Bc} &= \omega_i^{Bc} - \lambda_i^v (1-\tau_3) V_i^{Bc} \\
\dot{E}_i^{vn} &= \lambda_i^v (1-\tau_1) V_i^{Fn} + \lambda_i^v (1-\tau_2) V_i^{Sn} + \lambda_i^v (1-\tau_3) V_i^{Bn} - \alpha E_i^{vn} \\
\dot{E}_i^{vc} &= \lambda_i^v (1-\tau_1) V_i^{Fc} + \lambda_i^v (1-\tau_2) V_i^{sc} + \lambda_i^v (1-\tau_3) V_i^{Bc} - \alpha E_i^{vc} \\
\dot{A}_i^{vn} &= \alpha(1-\rho^v) E_i^{vn} - \gamma^a A_i^{vn} \\
\dot{A}_i^{vc} &= \alpha(1-\rho^v) E_i^{vc} - \gamma^a A_i^{vc} \\
\dot{I}_i^{vn} &= \alpha\rho^v E_i^{vn} - q I_i^{vn} \\
\dot{I}_i^{vc} &= \alpha\rho^v E_i^{vc} - q I_i^{vc} \\
\dot{H}_i^{mvn} &= q(1-\kappa_i^v) I_i^{vn} - \gamma_i H_i^{mvn} \\
\dot{H}_i^{mvc} &= q(1-\kappa_i^v) I_i^{vc} - \gamma_i H_i^{mvc} \\
\dot{H}_i^{svn} &= q\kappa_i^v I_i^{vn} - \gamma_i H_i^{svn} \\
\dot{H}_i^{svc} &= q\kappa_i^v I_i^{vc} - \gamma_i H_i^{svc} \\
\dot{R}_i^v &= \gamma^a (A_i^{vn} + A_i^{vc}) + \gamma_i (H_i^{mvn} + H_i^{mvc}) + \gamma_i (1-\delta_i^v)(H_i^{svn} + H_i^{svc}) \\
\dot{R}_i^{V^F} &= \omega_i^{FR} - \omega_i^{SR} \\
\dot{R}_i^{V^S} &= \omega_i^{SR}
\end{aligned}
\tag{S1}
$$

where

$$
\begin{aligned}
\lambda_i =& \beta_i \sum_{j=1}^{8} \left[\frac{m_{ij}(\theta_A(A_j^n + A_j^c + A_j^{vn} + A_j^{vc}) + (I_j^n + I_j^c + I_j^{vn} + I_j^{vc}))}{\tilde{N}_j} \right] \\
\lambda_i^v =& \beta_i^v \sum_{j=1}^{8} \left[\frac{m_{ij}^v(\theta_A(A_j^n + A_j^c + A_j^{vn} + A_j^{vc}) + (I_j^n + I_j^c + I_j^{vn} + I_j^{vc}))}{\tilde{N}_j} \right], \text{ and} \\
\tilde{N}_j =& S_j^n + S_j^c + E_j^n + E_j^c + A_j^n + A_j^c + I_j^n + I_j^c + R_j + V_j^{Fn} + V_j^{Fc} + V_j^{Sn} + V_j^{sc} \\
& + V_j^{Bn} + V_j^{Bc} + E_j^{vn} + E_j^{vc} + A_j^{vn} + A_j^{vc} + I_j^{vn} + I_j^{vc} + R_j^v + R_j^{V^F} + R_j^{V^S}.
\end{aligned} \tag{S2}
$$

Table S1. Parameter definitions and baseline values that were used in numerical simulations

Parameter	Description	Value	Ref.
β_i	Infection probability of an unvaccinated person in age group i per contact	Vary	Estimated
β_i^v	Infection probability of a vaccinated person without antibody in age group i per contact	Vary	Estimated
m_{ij}	Number of contacts made by a person in age group j with people in age group i	Vary	(1)
θ_A	Relative infectiousness of asymptomatic infectious state	0.51	(1)
τ_1	The first dose vaccine efficacy	0.61	(2)
τ_2	The second dose vaccine efficacy	0.9, vary	(3)
τ_3	The third dose vaccine efficacy	0.755, vary	(4)
$1/\alpha$	Latent period (day)	3.3	(5)
ρ	Probability of confirmed and infectious of unvaccinated cases	0.0875	(6)
ρ^v	Probability of confirmed and infectious of vaccinated cases	0.1319	Assumed, (6)
$1/q$	Mean duration of the case confirmation (day)	3	(7)
κ_i^n	Probability of an unvaccinated individual in normal condition having severe symptom	.0002, 0.0003, 0.0018, 0.0046, 0.0093, 0.0173, 0.0257, 0.0501	(8; 9)
κ_i^c	Probability of an unvaccinated individual with comorbities having severe symptom	0.0007, 0.0011, 0.0067, 0.0170, 0.0344, 0.0640, 0.0951, 0.1854	(8; 9)
κ_i^v	Probability of an unvaccinated individual having severe symptom	0, 0, 0.0011, 0.0062, 0.00466, 0.0362, 0.0125, 0.0385	(8)
$1/\gamma_i$	Mean period until discharge or death in age group i (day)	13.29, 11.40, 14.45, 14.31, 14.78, 15.76, 17.66, 22.96	Estimated
$1/\gamma^a$	Recovery period of asymptomatic case (day)	8	(10; 11)
$1/\gamma$	Recovery period of individuals with sever symptom (day)		
μ_i	Probability of death of unvaccinated patients in age group i	0 , 0, .0001, 0.0003, 0.0004, 0.0025, 0.0101, 0.0318, 0.0625	(12)
μ_i^v	Probability of death of vaccination completed patients in age group i	0, 0, 0, 0, 0, 0, 0, 0.0055	(12)
δ_i	Probability of death of H^s in age group i	μ_i/κ_i	Estimated
δ_i^v	Probability of death of H^{sv} in age group i	μ_i^v/κ_i^v	(2)
η_i	Proportion of individuals having at least one underlying medical condition in age group i	0.143, 0.094, 0.138, 0.206, 0.302, 0.404, 0.609, 0.801	Table 1
ξ	Probability of decedents having at least one underlying medical condition	0.886	(13)
δ_i^n	Probability of death of H^{sn} in age group i	$\frac{(1-\xi)\mu_i}{(1-\eta_i)\kappa_i}$	Estimated
δ_i^c	Probability of death of H^{sc} in age group i	$\frac{\xi\mu_i}{\eta_i\kappa_i}$	Estimated
$1/\psi$	Mean period between the first and the second dose of vaccination (day)	39	(14)

Parameter	Description	Value	Ref.
ω_i^{Fn}	Daily first dose of susceptibles without comorbid disease in age group i	$\nu^{Fn}\phi^{Fn}$	
ω_i^{Fc}	Daily first dose of susceptibles with comorbid disease in age group i	$\nu^{Fc}\phi^{Fc}$	
ω_i^{FR}	Daily first dose of unvaccinated recovered populations in age group i	$\nu^{FR}\phi^{FR}$	
ω_i^{Sn}	Daily second dose of susceptibles without comorbid disease in age group i	ψV_i^{Fn}	
ω_i^{Sc}	Daily second dose of susceptibles with comorbid disease in age group i	ψV_i^{Fc}	
ω_i^{SR}	Daily second dose of unvaccinated recovered populations in age group i	ψV_i^{FR}	
ω_i^{Bn}	Daily third dose of susceptibles without comorbid disease in age group i	$\nu^{Bn}\phi^{Bn}$	
ω_i^{Bc}	Daily third dose of susceptibles with comorbid disease in age group i	$\nu^{Bc}\phi^{Bc}$	
ν_0	Daily vaccination doses	10000	Assumed, (14)
ν^F	Daily first vaccination doses	$\nu_0 - \sum \omega_i^{Sn} - \sum \omega_i^{Sc} - \sum \omega_i^{SR}$	
ν^{Fn}	Daily first dose of susceptibles without comorbid disease	$\frac{\nu^F \sum_i S_i^n}{\sum_i S_i^n + \sum_i S_i^c + \sum_i R_i}$	
ν^{Fc}	Daily first dose of susceptibles with comorbid disease	$\frac{\nu^F \sum_i S_i^c}{\sum_i S_i^n + \sum_i S_i^c + \sum_i R_i}$	
ν^{FR}	Daily first dose of sunvaccinated recovered populations	$\frac{\nu^F \sum_i R_i}{\sum_i S_i^n + \sum_i S_i^c + \sum_i R_i}$	
ϕ_i^{Fn}	first vaccination allocation of susceptibles without comorbid disease in age group i (* represents age groups to be vaccinated according to the vaccination strategy)	$S_i^{n*} / \sum_i S_i^{n*}$	
ϕ_i^{Fc}	first vaccination allocation of susceptibles with comorbid disease in age group i (* represents age groups to be vaccinated according to the vaccination strategy)	$S_i^{c*} / \sum_i S_i^{c*}$	
ϕ_i^{FR}	first vaccination allocation of unvaccinated recovered populations in age group i (* represents age groups to be vaccinated according to the vaccination strategy)	$R_i^* / \sum_i R_i^*$	
ν^B	Daily third vaccination doses	10000,vary	Assumed, (14)
ν^{Bn}	Daily third doses for population without comorbid disease	$\frac{\nu^B \sum_i V_i^{Sn}}{\sum_i V_i^{Sn} + \sum_i V_i^{Sc}}$	
ν^{Bc}	Daily third doses for population with comorbid disease	$\frac{\nu^B \sum_i V_i^{Sc}}{\sum_i V_i^{Sn} + \sum_i V_i^{Sc}}$	
ϕ_i^{Bn}	third vaccination allocation for population without comorbid disease in age group i (* represents age groups to be vaccinated according to the vaccination strategy)	$V_i^{Sn*} / \sum_i V_i^{Sn*}$	
ϕ_i^{Bc}	third vaccination allocation for population without comorbid disease in age group i (* represents age groups to be vaccinated according to the vaccination strategy)	$V_i^{Sc*} / \sum_i V_i^{Sc*}$	

2 EFFECTIVE REPRODUCTION NUMBER (R_T)

We computed the effective reproduction numbers for the model (1) in the main manuscript.
Let $\mathbf{x} = (E_i^n, A_i^n, I_i^n, E_i^c, A_i^c, I_i^c, E_i^{vn}, A_i^{vn}, I_i^{vn}, E_i^{vc}, A_i^{vc}, I_i^{vc})^T$ for $i = 1, 2, .., 8$. The system has the disease-free state $\mathbf{x_0} = \mathbf{0}$.
$F(\mathbf{x})$ represents all of the new infections. The net transition rates of the corresponding compartments are represented by $V(\mathbf{x})$. $F(\mathbf{x})$ and $V(\mathbf{x})$ are

$$F(\mathbf{x}) = \begin{pmatrix} \lambda_i S_i^n \\ \mathbf{0} \\ \mathbf{0} \\ \lambda_i S_i^c \\ \mathbf{0} \\ \mathbf{0} \\ \lambda_i^v(1-\tau_1)V_i^{Fn} + \lambda_i^v(1-\tau_2)V_i^{Sn} + \lambda_i^v(1-\tau_3)V_i^{Bn} \\ \mathbf{0} \\ \mathbf{0} \\ \lambda_i^v(1-\tau_1)V_i^{Fc} + \lambda_i^v(1-\tau_2)V_i^{Sc} + \lambda_i^v(1-\tau_3)V_i^{Bc} \\ \mathbf{0} \\ \mathbf{0} \end{pmatrix},$$

and

$$V(\mathbf{x}) = \begin{pmatrix} \alpha E_i^n \\ -(1-\rho)\alpha E_i^n + \gamma^a A_i^n \\ -\rho\alpha E_i^n + q I_i^n \\ \alpha E_i^c \\ -(1-\rho)\alpha E_i^c + \gamma^a A_i^c \\ -\rho\alpha E_i^c + q I_i^c \\ \alpha E_i^{vn} \\ -(1-\rho^v)\alpha E_i^{vn} + \gamma^a A_i^{vn} \\ -\rho^v\alpha E_i^{vn} + q I_i^{vn} \\ \alpha E_i^{vc} \\ -(1-\rho^v)\alpha E_i^{vc} + \gamma^a A_i^{vc} \\ -\rho^v\alpha E_i^{vc} + q I_i^{vc} \end{pmatrix}.$$

$\mathbf{F}$ and $\mathbf{V}$ are 96×96 matrices obtained by $\mathbf{F} = \frac{\partial \mathbf{F}}{\partial X}\Big|_{\mathbf{x}=\mathbf{x}_0}$ and $\mathbf{V} = \frac{\partial \mathbf{V}}{\partial X}\Big|_{\mathbf{x}=\mathbf{x}_0}$.

$$
\mathbf{F} = \begin{pmatrix}
\mathbf{0} & \theta_A M_A^n & M_A^n & \mathbf{0} & \theta_A M_A^n & M_A^n & \mathbf{0} & \theta_A M_A^n & M_A^n & \mathbf{0} & \theta_A M_A^n & M_A^n \\
\mathbf{0} & \mathbf{0} & \mathbf{0} & \mathbf{0} & \mathbf{0} & \mathbf{0} & \mathbf{0} & \mathbf{0} & \mathbf{0} & \mathbf{0} & \mathbf{0} & \mathbf{0} \\
\mathbf{0} & \mathbf{0} & \mathbf{0} & \mathbf{0} & \mathbf{0} & \mathbf{0} & \mathbf{0} & \mathbf{0} & \mathbf{0} & \mathbf{0} & \mathbf{0} & \mathbf{0} \\
\mathbf{0} & \theta_A M_A^c & M_A^c & \mathbf{0} & \theta_A M_A^c & M_A^c & \mathbf{0} & \theta_A M_A^c & M_A^c & \mathbf{0} & \theta_A M_A^c & M_A^c \\
\mathbf{0} & \mathbf{0} & \mathbf{0} & \mathbf{0} & \mathbf{0} & \mathbf{0} & \mathbf{0} & \mathbf{0} & \mathbf{0} & \mathbf{0} & \mathbf{0} & \mathbf{0} \\
\mathbf{0} & \mathbf{0} & \mathbf{0} & \mathbf{0} & \mathbf{0} & \mathbf{0} & \mathbf{0} & \mathbf{0} & \mathbf{0} & \mathbf{0} & \mathbf{0} & \mathbf{0} \\
\mathbf{0} & \theta_A M_B^n & M_B^n & \mathbf{0} & \theta_A M_B^n & M_B^n & \mathbf{0} & \theta_A M_B^n & M_B^n & \mathbf{0} & \theta_A M_B^n & M_B^n \\
\mathbf{0} & \mathbf{0} & \mathbf{0} & \mathbf{0} & \mathbf{0} & \mathbf{0} & \mathbf{0} & \mathbf{0} & \mathbf{0} & \mathbf{0} & \mathbf{0} & \mathbf{0} \\
\mathbf{0} & \mathbf{0} & \mathbf{0} & \mathbf{0} & \mathbf{0} & \mathbf{0} & \mathbf{0} & \mathbf{0} & \mathbf{0} & \mathbf{0} & \mathbf{0} & \mathbf{0} \\
\mathbf{0} & \theta_A M_B^c & M_B^c & \mathbf{0} & \theta_A M_B^c & M_B^c & \mathbf{0} & \theta_A M_B^c & M_B^c & \mathbf{0} & \theta_A M_B^c & M_B^c \\
\mathbf{0} & \mathbf{0} & \mathbf{0} & \mathbf{0} & \mathbf{0} & \mathbf{0} & \mathbf{0} & \mathbf{0} & \mathbf{0} & \mathbf{0} & \mathbf{0} & \mathbf{0} \\
\mathbf{0} & \mathbf{0} & \mathbf{0} & \mathbf{0} & \mathbf{0} & \mathbf{0} & \mathbf{0} & \mathbf{0} & \mathbf{0} & \mathbf{0} & \mathbf{0} & \mathbf{0}
\end{pmatrix}.
$$

where

$$
\begin{aligned}
M_A^n &= diag\{\beta_i S_i^n\}_8 * C_M * diag\{1/N_i\}_8, \\
M_A^c &= diag\{\beta_i S_i^c\}_8 * C_M * diag\{1/N_i\}_8, \\
M_B^n &= diag\{\beta_i^v(\lambda_i^v(1-\tau_1)V_i^{Fn} + \lambda_i^v(1-\tau_2)V_i^{Sn} + \lambda_i^v(1-\tau_3)V_i^{Bn})\}_8 * C_M^v * diag\{1/N_i\}_8, \\
M_B^c &= diag\{\beta_i^v(\lambda_i^v(1-\tau_1)V_i^{Fc} + \lambda_i^v(1-\tau_2)V_i^{Sc} + \lambda_i^v(1-\tau_3)V_i^{Bc})\}_8 * C_M^v * diag\{1/N_i\}_8,
\end{aligned}
$$

C_M and C_M^v represent the contact matrices for unvaccinated and vaccinated, and $diag\{k_i\}_8$ represents an 8×8 diagonal matrix such that the ii entry is k_i.

$$
\mathbf{V} = \begin{pmatrix}
\alpha\mathbf{I}_8 & \mathbf{0} & \mathbf{0} & \mathbf{0} & \mathbf{0} & \mathbf{0} & \mathbf{0} & \mathbf{0} & \mathbf{0} & \mathbf{0} & \mathbf{0} & \mathbf{0} \\
-(1-\rho)\alpha\mathbf{I}_8 & \gamma^a\mathbf{I}_8 & \mathbf{0} & \mathbf{0} & \mathbf{0} & \mathbf{0} & \mathbf{0} & \mathbf{0} & \mathbf{0} & \mathbf{0} & \mathbf{0} & \mathbf{0} \\
-\rho\alpha\mathbf{I}_8 & \mathbf{0} & q\mathbf{I}_8 & \mathbf{0} & \mathbf{0} & \mathbf{0} & \mathbf{0} & \mathbf{0} & \mathbf{0} & \mathbf{0} & \mathbf{0} & \mathbf{0} \\
\mathbf{0} & \mathbf{0} & \mathbf{0} & \alpha\mathbf{I}_8 & \mathbf{0} & \mathbf{0} & \mathbf{0} & \mathbf{0} & \mathbf{0} & \mathbf{0} & \mathbf{0} & \mathbf{0} \\
\mathbf{0} & \mathbf{0} & \mathbf{0} & -(1-\rho)\alpha\mathbf{I}_8 & \gamma^a\mathbf{I}_8 & \mathbf{0} & \mathbf{0} & \mathbf{0} & \mathbf{0} & & & \\
\mathbf{0} & \mathbf{0} & \mathbf{0} & -\rho\alpha\mathbf{I}_8 & \mathbf{0} & q\mathbf{I}_8 & \mathbf{0} & \mathbf{0} & \mathbf{0} & \mathbf{0} & \mathbf{0} & \mathbf{0} \\
\mathbf{0} & \mathbf{0} & \mathbf{0} & \mathbf{0} & \mathbf{0} & \mathbf{0} & \alpha\mathbf{I}_8 & \mathbf{0} & \mathbf{0} & \mathbf{0} & \mathbf{0} & \mathbf{0} \\
\mathbf{0} & \mathbf{0} & \mathbf{0} & \mathbf{0} & \mathbf{0} & \mathbf{0} & -(1-\rho^v)\alpha\mathbf{I}_8 & \gamma^a\mathbf{I}_8 & \mathbf{0} & \mathbf{0} & \mathbf{0} & \mathbf{0} \\
\mathbf{0} & \mathbf{0} & \mathbf{0} & \mathbf{0} & \mathbf{0} & \mathbf{0} & -\rho^v\alpha\mathbf{I}_8 & \mathbf{0} & q\mathbf{I}_8 & \mathbf{0} & \mathbf{0} & \mathbf{0} \\
\mathbf{0} & \mathbf{0} & \mathbf{0} & \mathbf{0} & \mathbf{0} & \mathbf{0} & \mathbf{0} & \mathbf{0} & \mathbf{0} & \alpha\mathbf{I}_8 & \mathbf{0} & \mathbf{0} \\
\mathbf{0} & \mathbf{0} & \mathbf{0} & \mathbf{0} & \mathbf{0} & \mathbf{0} & \mathbf{0} & \mathbf{0} & \mathbf{0} & -(1-\rho^v)\alpha\mathbf{I}_8 & \gamma^a\mathbf{I}_8 & \mathbf{0} \\
\mathbf{0} & \mathbf{0} & \mathbf{0} & \mathbf{0} & \mathbf{0} & \mathbf{0} & \mathbf{0} & \mathbf{0} & \mathbf{0} & -\rho^v\alpha\mathbf{I}_8 & \mathbf{0} & q\mathbf{I}_8
\end{pmatrix}.
$$

The inverse matrix of $\mathbf{V}$ is

$$\mathbf{V}^{-1} = \begin{pmatrix}
\frac{1}{\alpha}\mathbf{I}_8 & \mathbf{0} & \mathbf{0} & \mathbf{0} & \mathbf{0} & \mathbf{0} & \mathbf{0} & \mathbf{0} & \mathbf{0} & \mathbf{0} & \mathbf{0} & \mathbf{0} \\
\frac{1-\rho}{\gamma^a}\mathbf{I}_8 & \frac{1}{\gamma^a}\mathbf{I}_8 & \mathbf{0} & \mathbf{0} & \mathbf{0} & \mathbf{0} & \mathbf{0} & \mathbf{0} & \mathbf{0} & \mathbf{0} & \mathbf{0} & \mathbf{0} \\
\frac{\rho}{q}\mathbf{I}_8 & \mathbf{0} & \frac{1}{q}\mathbf{I}_8 & \mathbf{0} & \mathbf{0} & \mathbf{0} & \mathbf{0} & \mathbf{0} & \mathbf{0} & \mathbf{0} & \mathbf{0} & \mathbf{0} \\
\mathbf{0} & \mathbf{0} & \mathbf{0} & \frac{1}{\alpha}\mathbf{I}_8 & \mathbf{0} & \mathbf{0} & \mathbf{0} & \mathbf{0} & \mathbf{0} & \mathbf{0} & \mathbf{0} & \mathbf{0} \\
\mathbf{0} & \mathbf{0} & \mathbf{0} & \frac{1-\rho}{\gamma^a}\mathbf{I}_8 & \frac{1}{\gamma^a}\mathbf{I}_8 & \mathbf{0} & \mathbf{0} & \mathbf{0} & \mathbf{0} & \mathbf{0} & \mathbf{0} & \mathbf{0} \\
\mathbf{0} & \mathbf{0} & \mathbf{0} & \frac{\rho}{q}\mathbf{I}_8 & \mathbf{0} & \frac{1}{q}\mathbf{I}_8 & \mathbf{0} & \mathbf{0} & \mathbf{0} & \mathbf{0} & \mathbf{0} & \mathbf{0} \\
\mathbf{0} & \mathbf{0} & \mathbf{0} & \mathbf{0} & \mathbf{0} & \mathbf{0} & \frac{1}{\alpha}\mathbf{I}_8 & \mathbf{0} & \mathbf{0} & \mathbf{0} & \mathbf{0} & \mathbf{0} \\
\mathbf{0} & \mathbf{0} & \mathbf{0} & \mathbf{0} & \mathbf{0} & \mathbf{0} & \frac{1-\rho^v}{\gamma^a}\mathbf{I}_8 & \frac{1}{\gamma^a}\mathbf{I}_8 & \mathbf{0} & \mathbf{0} & \mathbf{0} & \mathbf{0} \\
\mathbf{0} & \mathbf{0} & \mathbf{0} & \mathbf{0} & \mathbf{0} & \mathbf{0} & \frac{\rho^v}{q}\mathbf{I}_8 & \mathbf{0} & \frac{1}{q}\mathbf{I}_8 & \mathbf{0} & \mathbf{0} & \mathbf{0} \\
\mathbf{0} & \mathbf{0} & \mathbf{0} & \mathbf{0} & \mathbf{0} & \mathbf{0} & \mathbf{0} & \mathbf{0} & \mathbf{0} & \frac{1}{\alpha}\mathbf{I}_8 & \mathbf{0} & \mathbf{0} \\
\mathbf{0} & \mathbf{0} & \mathbf{0} & \mathbf{0} & \mathbf{0} & \mathbf{0} & \mathbf{0} & \mathbf{0} & \mathbf{0} & \frac{1-\rho^v}{\gamma^a}\mathbf{I}_8 & \frac{1}{\gamma^a}\mathbf{I}_8 & \mathbf{0} \\
\mathbf{0} & \mathbf{0} & \mathbf{0} & \mathbf{0} & \mathbf{0} & \mathbf{0} & \mathbf{0} & \mathbf{0} & \mathbf{0} & \frac{\rho^v}{q}\mathbf{I}_8 & \mathbf{0} & \frac{1}{q}\mathbf{I}_8
\end{pmatrix}$$

Then, the next generation matrix is

$$\begin{aligned}
\mathbf{G} &= \mathbf{F}\mathbf{V}^{-1} \\
&= \begin{pmatrix}
\zeta M_A^n & \frac{\theta_A}{q} M_A^n & \frac{1}{q} M_A^n & \zeta M_A^n & \frac{\theta_A}{q} M_A^n & \frac{1}{q} M_A^n & \zeta^v M_A^n & \frac{\theta_A}{q} M_A^n & \frac{1}{q} M_A^n & \zeta^v M_A^n & \frac{\theta_A}{q} M_A^n & \frac{1}{q} M_A^n \\
\mathbf{0} & \mathbf{0} & \mathbf{0} & \mathbf{0} & \mathbf{0} & \mathbf{0} & \mathbf{0} & \mathbf{0} & \mathbf{0} & \mathbf{0} & \mathbf{0} & \mathbf{0} \\
\mathbf{0} & \mathbf{0} & \mathbf{0} & \mathbf{0} & \mathbf{0} & \mathbf{0} & \mathbf{0} & \mathbf{0} & \mathbf{0} & \mathbf{0} & \mathbf{0} & \mathbf{0} \\
\zeta M_A^c & \frac{\theta_A}{q} M_A^c & \frac{1}{q} M_A^c & \zeta M_A^c & \frac{\theta_A}{q} M_A^c & \frac{1}{q} M_A^c & \zeta^v M_A^c & \frac{\theta_A}{q} M_A^c & \frac{1}{q} M_A^c & \zeta^v M_A^c & \frac{\theta_A}{q} M_A^c & \frac{1}{q} M_A^c \\
\mathbf{0} & \mathbf{0} & \mathbf{0} & \mathbf{0} & \mathbf{0} & \mathbf{0} & \mathbf{0} & \mathbf{0} & \mathbf{0} & \mathbf{0} & \mathbf{0} & \mathbf{0} \\
\mathbf{0} & \mathbf{0} & \mathbf{0} & \mathbf{0} & \mathbf{0} & \mathbf{0} & \mathbf{0} & \mathbf{0} & \mathbf{0} & \mathbf{0} & \mathbf{0} & \mathbf{0} \\
\zeta M_B^n & \frac{\theta_A}{q} M_B^n & \frac{1}{q} M_B^n & \zeta M_B^n & \frac{\theta_A}{q} M_B^n & \frac{1}{q} M_B^n & \zeta^v M_B^n & \frac{\theta_A}{q} M_B^n & \frac{1}{q} M_B^n & \zeta^v M_B^n & \frac{\theta_A}{q} M_B^n & \frac{1}{q} M_B^n \\
\mathbf{0} & \mathbf{0} & \mathbf{0} & \mathbf{0} & \mathbf{0} & \mathbf{0} & \mathbf{0} & \mathbf{0} & \mathbf{0} & \mathbf{0} & \mathbf{0} & \mathbf{0} \\
\mathbf{0} & \mathbf{0} & \mathbf{0} & \mathbf{0} & \mathbf{0} & \mathbf{0} & \mathbf{0} & \mathbf{0} & \mathbf{0} & \mathbf{0} & \mathbf{0} & \mathbf{0} \\
\zeta M_B^c & \frac{\theta_A}{q} M_B^c & \frac{1}{q} M_B^c & \zeta M_B^c & \frac{\theta_A}{q} M_B^c & \frac{1}{q} M_B^c & \zeta^v M_B^c & \frac{\theta_A}{q} M_B^c & \frac{1}{q} M_B^c & \zeta^v M_B^c & \frac{\theta_A}{q} M_B^c & \frac{1}{q} M_B^c \\
\mathbf{0} & \mathbf{0} & \mathbf{0} & \mathbf{0} & \mathbf{0} & \mathbf{0} & \mathbf{0} & \mathbf{0} & \mathbf{0} & \mathbf{0} & \mathbf{0} & \mathbf{0} \\
\mathbf{0} & \mathbf{0} & \mathbf{0} & \mathbf{0} & \mathbf{0} & \mathbf{0} & \mathbf{0} & \mathbf{0} & \mathbf{0} & \mathbf{0} & \mathbf{0} & \mathbf{0}
\end{pmatrix},
\end{aligned}$$

where $\zeta = \left(\frac{\theta_A(1-\rho)}{\gamma^a} + \frac{\rho}{q}\right)$, and $\zeta^v = \left(\frac{\theta_A(1-\rho^v)}{\gamma^a} + \frac{\rho^v}{q}\right)$.

Finally, the effective reproduction number R_t is computed as the spectral radius $\rho(\mathbf{G})$ of the next generation matrix G.

3 CONTACT MATRICES

M^L, M^M and M^H represent the contact matrices used for NPI level low, moderate and high respectively.

$$M^L = \begin{bmatrix}
3.8124 & 1.0646 & 0.5137 & 1.8609 & 1.2177 & 0.5976 & 0.2866 & 0.1268 \\
1.2789 & 18.6651 & 1.8695 & 1.8084 & 3.1984 & 1.5658 & 0.3696 & 0.2889 \\
0.8095 & 2.4521 & 6.3691 & 3.3998 & 3.0077 & 2.2671 & 0.5384 & 0.1553 \\
2.8889 & 2.3368 & 3.3494 & 8.2011 & 4.8345 & 2.6878 & 1.2788 & 0.4229 \\
2.3169 & 5.0653 & 3.6315 & 5.9249 & 8.4655 & 3.9550 & 1.3361 & 0.8213 \\
1.2852 & 2.8031 & 3.0941 & 3.7236 & 4.4706 & 5.3080 & 1.3291 & 0.5977 \\
0.4852 & 0.5208 & 0.5784 & 1.3945 & 1.1888 & 1.0462 & 0.9238 & 0.2143 \\
0.2955 & 0.5605 & 0.2297 & 0.6350 & 1.0063 & 0.6478 & 0.2951 & 0.2669
\end{bmatrix}$$

$$M^M = \begin{bmatrix} 2.8955 & 0.9309 & 0.4895 & 1.8147 & 1.1644 & 0.5131 & 0.2815 & 0.1326 \\ 1.1184 & 12.6679 & 1.5371 & 1.5322 & 2.8437 & 1.2600 & 0.3310 & 0.2995 \\ 0.7713 & 2.0161 & 5.4112 & 2.5445 & 2.3302 & 1.9543 & 0.4827 & 0.1510 \\ 2.8172 & 1.9800 & 2.5068 & 6.8415 & 3.5436 & 2.0415 & 1.1566 & 0.4128 \\ 2.2154 & 4.5035 & 2.8135 & 4.3429 & 6.8624 & 2.9358 & 1.1267 & 0.8078 \\ 1.1036 & 2.2556 & 2.6672 & 2.8282 & 3.3186 & 4.6024 & 1.1562 & 0.5764 \\ 0.4765 & 0.4664 & 0.5185 & 1.2612 & 1.0025 & 0.9100 & 0.9181 & 0.2024 \\ 0.3091 & 0.5812 & 0.2234 & 0.6198 & 0.9898 & 0.6248 & 0.2788 & 0.2670 \end{bmatrix}$$

$$M^H = \begin{bmatrix} 2.4899 & 0.8953 & 0.4942 & 1.9536 & 1.2225 & 0.4649 & 0.3051 & 0.1555 \\ 1.0755 & 9.1443 & 1.4167 & 1.5256 & 2.9800 & 1.2114 & 0.3394 & 0.3459 \\ 0.7787 & 1.8583 & 5.6183 & 2.6370 & 2.5593 & 2.1750 & 0.5045 & 0.1499 \\ 3.0329 & 1.9715 & 2.5980 & 7.4018 & 3.7420 & 2.1095 & 1.1814 & 0.4112 \\ 2.3260 & 4.7194 & 3.0901 & 4.5860 & 7.6844 & 3.1162 & 1.0905 & 0.8264 \\ 0.9999 & 2.1686 & 2.9685 & 2.9224 & 3.5225 & 5.0776 & 1.1050 & 0.5742 \\ 0.5166 & 0.4782 & 0.5419 & 1.2882 & 0.9702 & 0.8698 & 0.9921 & 0.1867 \\ 0.3624 & 0.6711 & 0.2217 & 0.6175 & 1.0124 & 0.6224 & 0.2571 & 0.2853 \end{bmatrix}$$

4 RESULTS OF PARAMETER FITTING FOR β

Figure S1 compared cumulative confirmed cases with the model outputs of the cumulative confirmed cases fitting for all age groups for low, moderate and high NPI levels for the first, second and third vaccination duration in the given time interval.

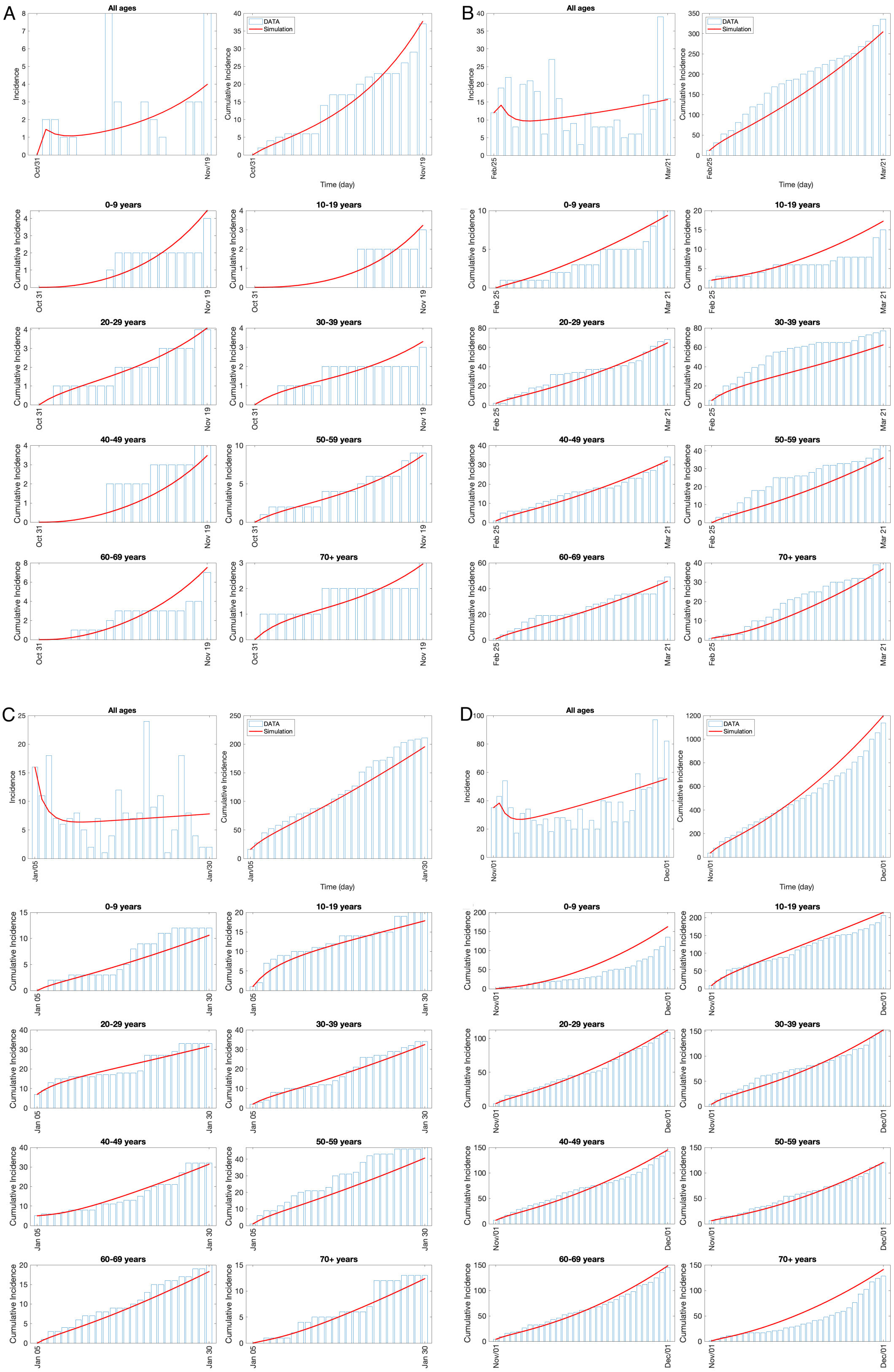

Figure S1. Results of data fitting for the transmission rate under **(A)** the low NPI level, **(B)** the moderate NPI level, **(C)** the high NPI level for the first and the second doses period, and **(D)** the low NPI level for the booster vaccination period.

5 VACCINATION EFFECTS

Table S2 shows the reduction rates of the number of cumulative cases and cumulative death for each NPI level, vaccination roll-out speed and vaccination strategies.

Table S2. Vaccination effects on the reduction rate of confirmed cases and death for various roll out speeds are shown. Bold texts indicate the most effective cases.

	NPI level	No vaccine (# of cases)	Daily doses	30 − 49	60+	Comorb.	20+
				(Reduction rates (%))			
Cumulative cases	Low	1.1290×10^6	5000	**26.044**	16.942	21.472	23.62
			7500	**37.533**	28.027	31.935	35.04
			10000	**47.912**	39.636	42.604	45.617
			15000	**64.601**	59.6	61.284	62.947
	Moderate	46113	5000	**94.525**	61.74	79.752	91.526
			7500	**97.207**	84.922	90.435	95.994
			10000	**98.167**	93.148	95.034	97.548
			15000	**98.89**	97.405	97.805	98.631
	High	1876.7	5000	**76.662**	29.978	55.021	64.862
			7500	**81.993**	50.178	65.95	73.535
			10000	**84.816**	63.004	72.796	78.357
			15000	**87.834**	75.593	80.173	83.516
Death	Low	8655.6	5000	22.294	**61.519**	56.204	34.034
			7500	37.235	74.447	**75.255**	48.954
			10000	53.56	81.273	**84.930**	62.025
			15000	78.507	90.677	**93.106**	82.019
	Moderate	320.28	5000	**73.275**	66.63	69.818	71.2
			7500	**76.539**	74.379	75.816	75.742
			10000	77.819	77.406	**77.863**	77.485
			15000	78.828	79.124	**79.192**	78.77
	High	72.915	5000	**9.5795**	7.6685	8.8245	8.4129
			7500	**10.47**	9.5034	10.327	9.7094
			10000	10.962	10.587	**11.114**	10.46
			15000	11.511	11.63	**11.903**	11.285

Table S3 shows the first and second vaccination effects on cumulative cases and mean of R_t for 60 days for each NPI level and vaccination strategies.

Table S3. The mean of R_t for 60 days are shown for each NPI level and vaccination strategies. Bold texts indicate the most effective cases.

NPI level	No vaccine	30 − 49	60+	Comorb.	20+
Low	1.9131	**1.7491**	1.8289	1.7787	1.7999
Moderate	1.2309	**0.9459**	1.1170	0.9804	1.0746
High	1.0515	**0.7875**	0.9728	0.8723	0.9728

The effects of the first and the second vaccination on each age groups for each vaccination strategy and NPI level are in Table S4, S5 and S6. The effects of the booster vaccination on each age groups for each vaccination strategy in Table S7.

Table S4. Vaccination effects on age groups with the low NPI level

	Age group	No vaccine	30 − 49	60+	Comorb.	20+
Cumulative cases	Total	1,129,000	588,080	681,520	648,010	613,990
	0-9	11,3470 (10.05%)	99,364 (16.89%)	106,880 (15.62%)	105,300 (16.24 %)	103,130 (16.79%)
	10-19	127,700 (11.31%)	113,970 (19.37%)	120,570 (17.69%)	118,690 (18.31%)	116,590 (18.98%)
	20-29	135,040 (11.96%)	65,334 (11.11%)	82,470 (12.10%)	77,346 (11.93%)	54,526 (8.880%)
	30-39	116,560 (10.32%)	13,830 (2.351%)	66,263 (9.723%)	59,604 (9.20%)	43,581 (7.10%)
	40-49	162,700 (14.41%)	27,661 (4.706%)	101,890 (14.95%)	85,255 (13.16%)	70,446 (11.47%)
	50-59	219,370 (19.43%)	126,480 (21.58%)	150,060 (22.02%)	111,510 (17.21 %)	108,460 (17.66%)
	60-69	180,580 (15.99%)	111,090 (18.89%)	43,148 (6.33%)	74,250 (11.46%)	91,505 (14.90%)
	70+	73,590 (6.52%)	30,359 (5.16%)	10,248 (1.50%)	16,051 (2.48%)	25,751 (4.19%)
Death	Total	8,656	4,020	1,621	1,304	3,287
	40-49	115.43 (1.33%)	11.397 (0.28%)	68.177 (4.21%)	18.452 (1.41%)	44.591 (1.36%)
	50-59	1,022.2 (11.81%)	540.8 (13.45%)	637.3 (39.32%)	162.9 (12.49%)	420.7 (12.8%)
	60-69	2,533.7 (29.27%)	1,480.1 (36.82%)	324.8 (20.04%)	438.6 (33.63%)	1,148.7 (34.95%)
	70+	4,884.4 (56.43%)	1,967.9 (48.96%)	535.3 (33.02%)	670. (51.36%)	1,638 (49.83%)

Table S5. Vaccination effects on age groups with the moderate NPI level

	Age group	No vaccine	30 − 49	60+	Comorb.	20+
Cumulative cases	Total	46,113.	845.18	3,159.9	2,289.9	1,130.7
	0 - 9	1,582.	32.65	153.25	107.66	51.83
		(3.43 %)	(3.86 %)	(4.85 %)	(4.7 %)	(4.58 %)
	10 - 19	2,550.3	58.32	264.49	182.57	85.08
		(5.53 %)	(6.9 %)	(8.37 %)	(7.97 %)	(7.52 %)
	20 - 29	11,265.	253.75	877.56	625.18	249.62
		(24.43 %)	(30.02 %)	(27.77 %)	(27.3 %)	(22.08 %)
	30 - 39	8,078.8	74.59	607.35	418.26	184.62
		(17.52 %)	(8.83 %)	(19.22 %)	(18.27 %)	(16.33 %)
	40 - 49	5,491.8	59.46	431.22	276.07	131.18
		(11.91 %)	(7.04 %)	(13.65 %)	(12.06 %)	(11.6 %)
	50 - 59	6,277.2	137.91	518.72	311.97	162.09
		(13.61 %)	(16.32 %)	(16.42 %)	(13.62 %)	(14.34 %)
	60 - 69	6,674.3	140.14	214.71	251.98	164.95
		(14.47 %)	(16.58 %)	(6.8 %)	(11. %)	(14.59 %)
	70 +	4,194.1	88.36	92.58	116.21	101.38
		(9.1 %)	(10.46 %)	(2.93 %)	(5.07 %)	(8.97 %)
Death	Total	320.28	71.04	72.37	70.9	72.11
	40 - 49	3.7	1.05	1.3	1.09	1.1
		(1.16 %)	(1.48 %)	(1.79 %)	(1.54 %)	(1.53 %)
	50 - 59	23.08	3.83	5.29	3.78	3.85
		(7.21 %)	(5.39 %)	(7.31 %)	(5.33 %)	(5.33 %)
	60 - 69	71.95	12.49	12.22	12.41	12.68
		(22.46 %)	(17.59 %)	(16.89 %)	(17.5 %)	(17.58 %)
	70 +	216.34	53.52	52.97	53.42	54.28
		(67.55 %)	(75.34 %)	(73.19 %)	(75.34 %)	(75.27 %)

Table S6. Vaccination effects on age groups with the high NPI level

	Age group	No vaccine	30-49	60+	Comorb.	20+
Cumulative cases	Total	1,876.7	284.95	694.3	510.53	406.17
	0-9	126.96 (6.77 %)	21.82 (7.66 %)	56.02 (8.07 %)	41.49 (8.13 %)	32.54 (8.01 %)
	10-19	133.83 (7.13 %)	21.17 (7.43 %)	58.91 (8.48 %)	42.05 (8.24 %)	32.38 (7.97 %)
	20-29	211.71 (11.28 %)	37.87 (13.29 %)	83.85 (12.08 %)	62.38 (12.22 %)	44.78 (11.02 %)
	30-39	345.92 (18.43 %)	38.97 (13.68 %)	133.33 (19.2 %)	97.64 (19.12 %)	71.14 (17.52 %)
	40-49	330.35 (17.6 %)	34.23 (12.01 %)	125.68 (18.1 %)	85.44 (16.74 %)	64.89 (15.98 %)
	50-59	409.32 (21.81 %)	67.33 (23.63 %)	158.34 (22.81 %)	101.12 (19.81 %)	83.85 (20.64 %)
	60-69	213.29 (11.37 %)	40.48 (14.21 %)	54.97 (7.92 %)	55.11 (10.8 %)	50.08 (12.33 %)
	70+	105.3 (5.61 %)	23.08 (8.1 %)	23.2 (3.34 %)	25.3 (4.96 %)	26.52 (6.53 %)
Death	Total	72.92	64.92	65.2	64.81	65.29
	40-49	1.22 (1.67 %)	1.04 (1.6 %)	1.1 (1.69 %)	1.05 (1.62 %)	1.06 (1.62 %)
	50-59	4.85 (6.66 %)	3.52 (5.43 %)	3.91 (5.99 %)	3.49 (5.38 %)	3.57 (5.46 %)
	60-69	13.11 (17.99 %)	11.13 (17.14 %)	11.05 (16.95 %)	11.09 (17.12 %)	11.21 (17.17 %)
	70+	53.46 (73.32 %)	49.15 (75.71 %)	48.99 (75.15 %)	49.09 (75.74 %)	49.35 (75.59 %)

Table S7. Booster vaccination effects on age groups with the low NPI level

	Age group	No vaccine	30-49	60+	Comorb.	20+
Cumulative cases	Total	188,810	99,463	120,450	105,450	100,050
	0-9	37,051 (19.62 %)	23,635 (23.76 %)	28,283 (23.48 %)	25,196 (23.9 %)	23,979 (23.97 %)
	10-19	139,572 (20.96 %)	27,120 (27.27 %)	31,774 (26.38 %)	28,650 (27.17 %)	27,430 (27.42 %)
	20-29	17,156 (9.09 %)	7,724.7 (7.77 %)	9,982.2 (8.29 %)	8,677.4 (8.23 %)	7,645.7 (7.64 %)
	30-39	13,569 (7.19 %)	6,885.6 (6.92 %)	8,826.5 (7.33 %)	7,741.5 (7.34 %)	7,153.9 (7.15 %)
	40-49	20,553 (10.89 %)	8,471 (8.52 %)	11,861 (9.85 %)	10,087 (9.57 %)	9,215 (9.21 %)
	50-59	32,849 (17.4 %)	12,272 (12.34 %)	15,759 (13.08 %)	12,218 (11.59 %)	11,661 (11.66 %)
	60-69	19,565 (10.36 %)	8,456. (8.5 %)	8,420.9 (6.99 %)	7,908.2 (7.5 %)	8,081.3 (8.08 %)
	70+	8,490.3 (4.5 %)	4,899.8 (4.93 %)	5,540.2 (4.6 %)	4,969.6 (4.71 %)	4,885 (4.88 %)
Death	Total	465.83	319.84	370.45	334.17	322.34
	40-49	4.01 (.86 %)	2.88 (.9 %)	3.28 (.89 %)	3.01 (.9 %)	2.91 (.9 %)
	50-59	17.28 (3.71 %)	11.72 (3.67 %)	13.58 (3.67 %)	12.24 (3.66 %)	11.78 (3.66 %)
	60-69	61.69 (13.24 %)	45.1 (14.1 %)	50.93 (13.75 %)	46.77 (13.99 %)	45.32 (14.06 %)
	70+	379.83 (81.54 %)	258.28 (80.75 %)	300.4 (81.09 %)	270.16 (80.85 %)	260.45 (80.8 %)

Table S8, S9 show the effect of vaccination during the simulation period of 1 year.

Table S8. Vaccination effects on the number of confirmed cases and deaths for various rollout speeds are shown. Bold texts indicate the most effective cases.

	NPI level	No vaccine (# of cases)	Daily doses	30 − 49	60+	Comorb. (# of cases)	20+
Cumulative cases	Low	1.1291×10^6	5000	**835,130**	937,830	886,730	862,490
			7500	**705,440**	812,660	768,590	733,560
			10000	**588,250**	681,580	648,100	614,110
			15000	**399,830**	456,180	437,180	418,430
	Moderate	480040	5000	**2,762.2**	39,407. 0	18,597.0	4,692.9
			7500	**1,300.5**	7,874.7	4,894.9	1,888.4
			10000	**846.08**	3,202.4	2,314.7	1,133.5
			15000	**511.85**	1,196.9	1,012.5	631.55
	High	8839	5000	**439.4**	1,629.5	927.65	680.8
			7500	**338.13**	967.03	649.88	499.41
			10000	**284.99**	697.83	511.96	406.6
			15000	**228.32**	458.13	372.15	309.37
Death	Low	8887	5000	6,947.2	**3,399.2**	3,892.3	5,882.8
			7500	5,617.6	2,256.0	**2,200.2**	4,556.3
			10000	4,160.2	1,652.2	**1,340.7**	3,389.2
			15000	1,916.1	820.2	**610.58**	1,597.4
	Moderate	4604	5000	**90.15**	176.83	124.56	102.7
			7500	**75.66**	86.60	79.77	78.74
			10000	**71.15**	72.81	71.19	72.30
			15000	67.83	66.89	**66.67**	68.02
	High	112	5000	**65.98**	68.36	66.75	67.04
			7500	**65.3 0**	66.14	65.44	65.89
			10000	64.93	65.23	**64.83**	65.31
			15000	64.53	64.44	**64.24**	64.69

Table S9. Vaccination effects on the reduction rate of confirmed cases and death for various roll out speeds are shown. Bold texts indicate the most effective cases.

	NPI level	No vaccine (# of cases)	Daily doses	30 − 49	60+	Comorb.	20+
				(Reduction rates (%))			
Cumulative cases	Low	1.1291×10^6	5000	**26.04**	16.94	21.47	23.61
			7500	**37.52**	28.03	31.93	35.03
			10000	**47.9**	39.64	42.6	45.61
			15000	**64.59**	59.6	61.28	62.94
	Moderate	480040	5000	**99.43**	91.79	96.13	99.02
			7500	**99.73**	98.36	98.98	99.61
			10000	**99.82**	99.33	99.52	99.76
			15000	**99.89**	99.75	99.79	99.87
	High	8839	5000	**95.03**	81.57	89.51	92.3
			7500	**96.18**	89.06	92.65	94.35
			10000	**96.78**	92.11	94.21	95.4
			15000	**97.42**	94.82	95.79	96.5
Death	Low	8887	5000	21.82	**61.75**	56.2	33.8
			7500	36.79	74.61	**75.24**	48.73
			10000	53.19	81.41	**84.91**	61.86
			15000	78.44	90.77	**93.13**	82.02
	Moderate	4604	5000	**98.04**	96.16	97.3	97.77
			7500	**98.36**	98.12	98.27	98.29
			10000	**98.46**	98.42	98.45	98.43
			15000	98.53	98.55	**98.55**	98.52
	High	112	5000	**41.32**	39.2	40.64	40.38
			7500	**41.92**	41.17	41.8	41.4
			10000	42.25	41.99	**42.34**	41.92
			15000	42.61	42.69	**42.86**	42.46

6 SENSITIVITY ANALYSIS

The sensitivity analyses show the relative importance of the parameters related to vaccination, which are β, β^v, ρ, ρ^v, τ_1, τ_2, ν_0, and $1/\psi$ in regard to the disease transmission dynamics. We performed sensitivity analysis on the model parameters described in Table 2 in the main manuscript. We used the normalized forward sensitivity index of the cumulative incidence (CI) on the parameter p, which was defined as $CI_p = \frac{\partial(CI)}{\partial p} \times \frac{p}{CI}$ (15). The CI for 1 year computed after varying one parameter by 5% from the baseline value while the rest of the parameters are fixed at the baseline values. For the vector parameter β and $\beta^{\mathbf{v}}$, all components were increased simultaneously. Table S10 shows that the increase in β and $\beta^{\mathbf{v}}$ affected the CI for all cases; however, the increase in τ_1, τ_2, ν_0, and $1/\psi$ negatively affected the CI for all cases. Furthermore, β is shown to be the most sensitive parameter, and this sensitivity was increased under a moderate NPI level, which was the case when vaccination was most effective. For the ρ, ρ^v, the sensitivity indices were both positive and negative, depending on the NPI level and vaccination scenarios. This could be interpreted a scenario wherein, at a low NPI level with many infections, the number of confirmed cases increase when the confirmation rate is increased. However, at a moderate or high NPI level with a relatively small number of infections, a decrease in unconfirmed asymptomatic infections decreases the total number of infections.

Table S10. Sensitivity analysis index for each NPI level and vaccination strategy

p	Low NPI				Moderate NPI				Nigh NPI			
	30-49	60 +	Comorb.	20+	30 - 49	60 +	Comorb.	20+	30 - 49	60 +	Comorb	20+
β	2.188	2.014	2.042	2.127	10.642	19.835	17.99	12.449	5.234	11.613	9.434	7.467
$\beta^{\mathbf{v}}$	0.251	0.223	0.228	0.252	0.732	0.715	0.769	0.771	0.378	0.287	0.347	0.367
ρ	0.769	0.785	0.775	0.759	-0.645	-1.451	-1.361	-0.855	-0.061	-0.974	-0.703	-0.428
ρ^v	0.098	0.13	0.12	0.117	-0.039	0.015	-0.009	-0.027	-0.003	0.02	0.007	0.001
τ_1	-0.289	-0.259	-0.261	-0.286	-0.918	-0.878	-0.933	-0.952	-0.503	-0.371	-0.447	-0.479
τ_2	-0.576	-0.516	-0.55	-0.613	-0.83	-0.952	-1.084	-0.97	-0.332	-0.337	-0.418	-0.4
ν_0	-0.694	-0.712	-0.684	-0.685	-1.345	-2.557	-2.151	-1.525	-0.544	-1.021	-0.764	-0.651
$1/\psi$	-0.034	-0.073	-0.047	-0.033	-0.067	-0.467	-0.208	-0.088	-0.003	-0.187	-0.053	-0.029

REFERENCES

[1]Choi Y, Kim JS, Kim JE, Choi H, Lee CH. Vaccination Prioritization Strategies for COVID-19 in Korea: A Mathematical Modeling Approach. International journal of environmental research and public health. 2021;18(8):4240.

[2]Bernal JL, Andrews N, Gower C, Robertson C, Stowe J, Tessier E, et al. Effectiveness of the Pfizer-BioNTech and Oxford-AstraZeneca vaccines on covid-19 related symptoms, hospital admissions, and mortality in older adults in England: test negative case-control study. bmj. 2021;373.

[3]Knoll MD, Wonodi C. Oxford–AstraZeneca COVID-19 vaccine efficacy. The Lancet. 2021;397(10269):72–74.

[4]Andrews N, Stowe J, Kirsebom F, Toffa S, Rickeard T, Gallagher E, et al. Effectiveness of COVID-19 vaccines against the Omicron (B. 1.1. 529) variant of concern. MedRxiv. 2021;.

[5]Zhao S, Tang B, Musa SS, Ma S, Zhang J, Zeng M, et al. Estimating the generation interval and inferring the latent period of COVID-19 from the contact tracing data. Epidemics. 2021;p. 100482.

[6]Lee C, Apio C, Park T. Estimation of Undetected Asymptomatic COVID-19 Cases in South Korea Using a Probabilistic Model. International Journal of Environmental Research and Public Health. 2021;18(9):4946.

[7]Choi Y, Kim JS, Choi H, Lee H, Lee CH. Assessment of Social Distancing for Controlling COVID-19 in Korea: An Age-Structured Modeling Approach. International journal of environmental research and public health. 2020;17(20):7474.

[8]; 2021. [Online; accessed 21-Feb-2022]. `http://ncov.mohw.go.kr/tcmBoardView.do?brdId=3&brdGubun=31&dataGubun=&ncvContSeq=5855&contSeq=5855&board_id=312&gubun=ALL`.

[9]Jain V, Yuan JM. Systematic review and meta-analysis of predictive symptoms and comorbidities for severe COVID-19 infection. Medrxiv. 2020;.

[10]Kissler SM, Tedijanto C, Goldstein E, Grad YH, Lipsitch M. Projecting the transmission dynamics of SARS-CoV-2 through the postpandemic period. Science. 2020;368(6493):860–868.

[11]Blyuss KB, Kyrychko YN. Effects of latency and age structure on the dynamics and containment of COVID-19. Journal of Theoretical Biology. 2021;513:110587.

[12]; 2021. [Online; accessed 21-Feb-2022]. `http://ncov.mohw.go.kr/tcmBoardView.do?brdId=3&brdGubun=31&dataGubun=&ncvContSeq=5953&contSeq=5953&board_id=312&gubun=ALL`.

[13]An C, Lim H, Kim DW, Chang JH, Choi YJ, Kim SW. Machine learning prediction for mortality of patients diagnosed with COVID-19: a nationwide Korean cohort study. Scientific reports. 2020;10(1):1–11.

[14]; 2022. [Online; accessed 16-February-2022]. https://ncv.kdca.go.kr/vaccineStatus.es?mid=a11710000000.

[15]Rodrigues HS, Monteiro MTT, Torres DF. Sensitivity analysis in a dengue epidemiological model. In: Conference papers in science. vol. 2013. Hindawi; 2013. .
